# Supplementary material for: Cellular immunotherapy targeting CLL-1 for juvenile myelomonocytic leukemia
Source: Nat Commun. 2025 Apr 23;16:3804. doi: 10.1038/s41467-025-59040-6 (PMC12019388; doi:10.1038/s41467-025-59040-6)
Supplement: Supplementary file 1 — Supplementary Information [file 41467_2025_59040_MOESM1_ESM.pdf]

Supplementary Information for

Cellular immunotherapy targeting CLL-1 for juvenile myelomonocytic leukemia

J. Werner and A. G. Lee et al.

Contents:

Supplementary Figures 1-29

Supplementary Fig. 1

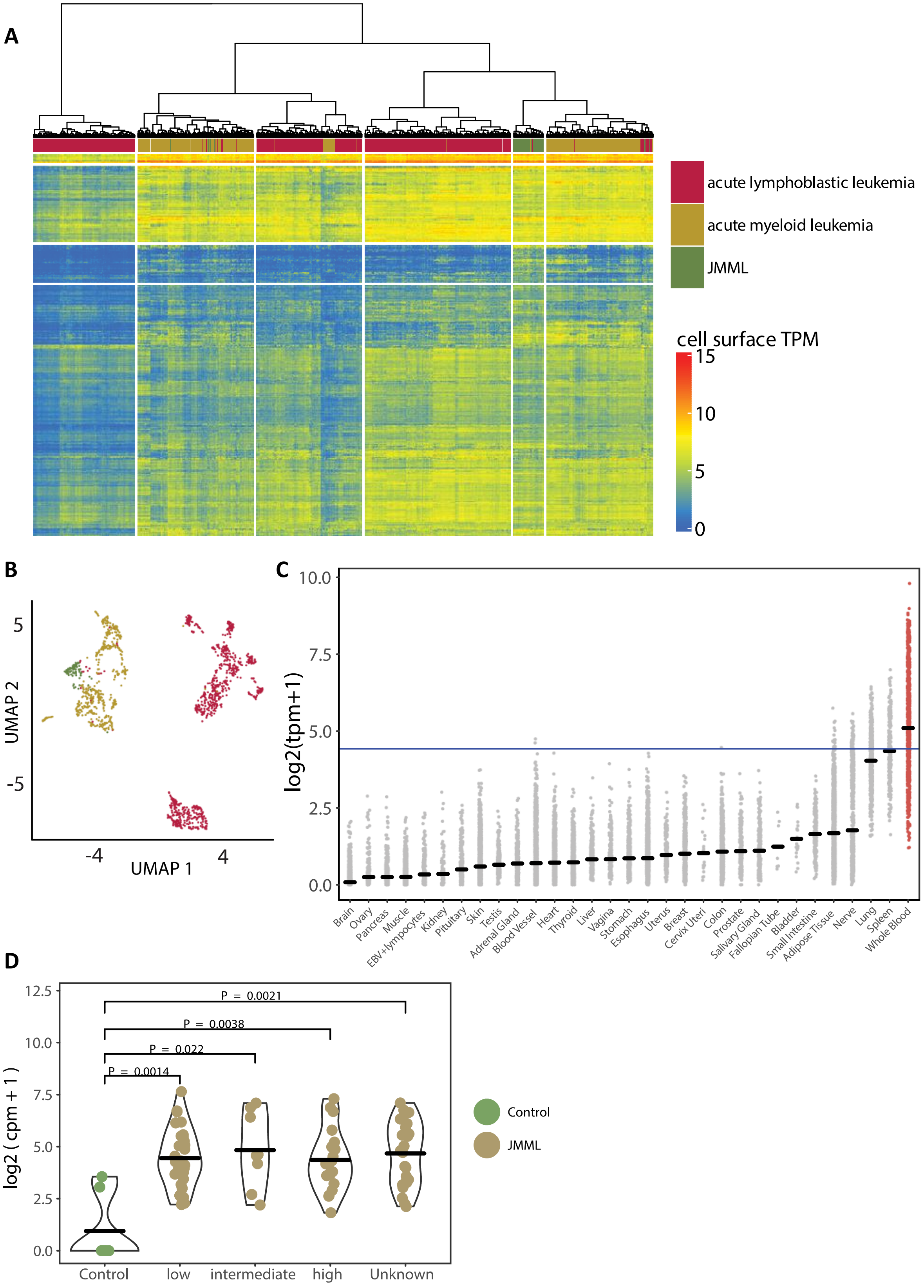

Supplementary Fig. 1: Bulk RNAseq uncovers unique cell surface signatures on JMML.

(A) Heatmap showing hierarchical clustering of surface protein encoding genes from bulk RNAseq comparing to pediatric AML and ALL RNAseq datasets retrieved from UCSC Treehouse.

(B) UMAP plot based on surface protein encoding genes from (A).

(C) Transcript abundance of *CLEC12A* in healthy tissue as determined by GTEx database. The blue line indicates top 95% of the entire set.

(D) CLEC12A expression across JMML methylation groups and controls. Violin plots show the distribution of CLEC12A expression levels (log2(cpm+1)) in control and JMML samples categorized by methylation status (low, intermediate, high, and unknown). Each dot represents an individual sample. Black horizontal lines within the violins represent the median expression for each group. P-values from unpaired two-tailed Wilcoxon rank-sum tests, with Bonferroni correction.

EBV+lymphocytes=EBV-transformed lymphocytes

Supplementary Fig. 2

A

Cell.Type	Description	Marker.panel
HSCs	(Hematopoietic Stem Cells)	Lin−CD34+CD38−CD90+CD45RA−
HSC-aberrant	(Aberrant Hematopoietic Stem Cells, Louka 2021)	Lin−CD34+CD38−CD90+CD45RA+
MPPs	(Multipotent Progenitors)	Lin−CD34+CD38−CD90−CD45RA−
LMPPs	(Lymphoid Primed Multipotent Progenitors)	Lin−CD34+CD38−CD90−CD45RA+
CMPs	(Common Myeloid Progenitors)	Lin−CD34+CD38+CD123+CD45RA−
MEPs	(Megakaryocyte Erythroid Progenitors)	Lin−CD34+CD38+CD123−CD45RA−
GMPs	(Granulocyte Monocyte Progenitors)	Lin−CD34+CD38+CD123+CD45RA+

B

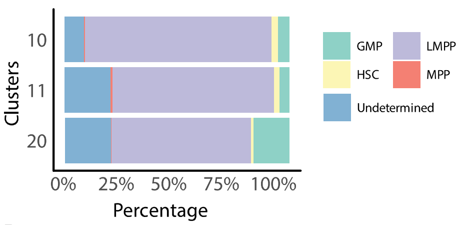

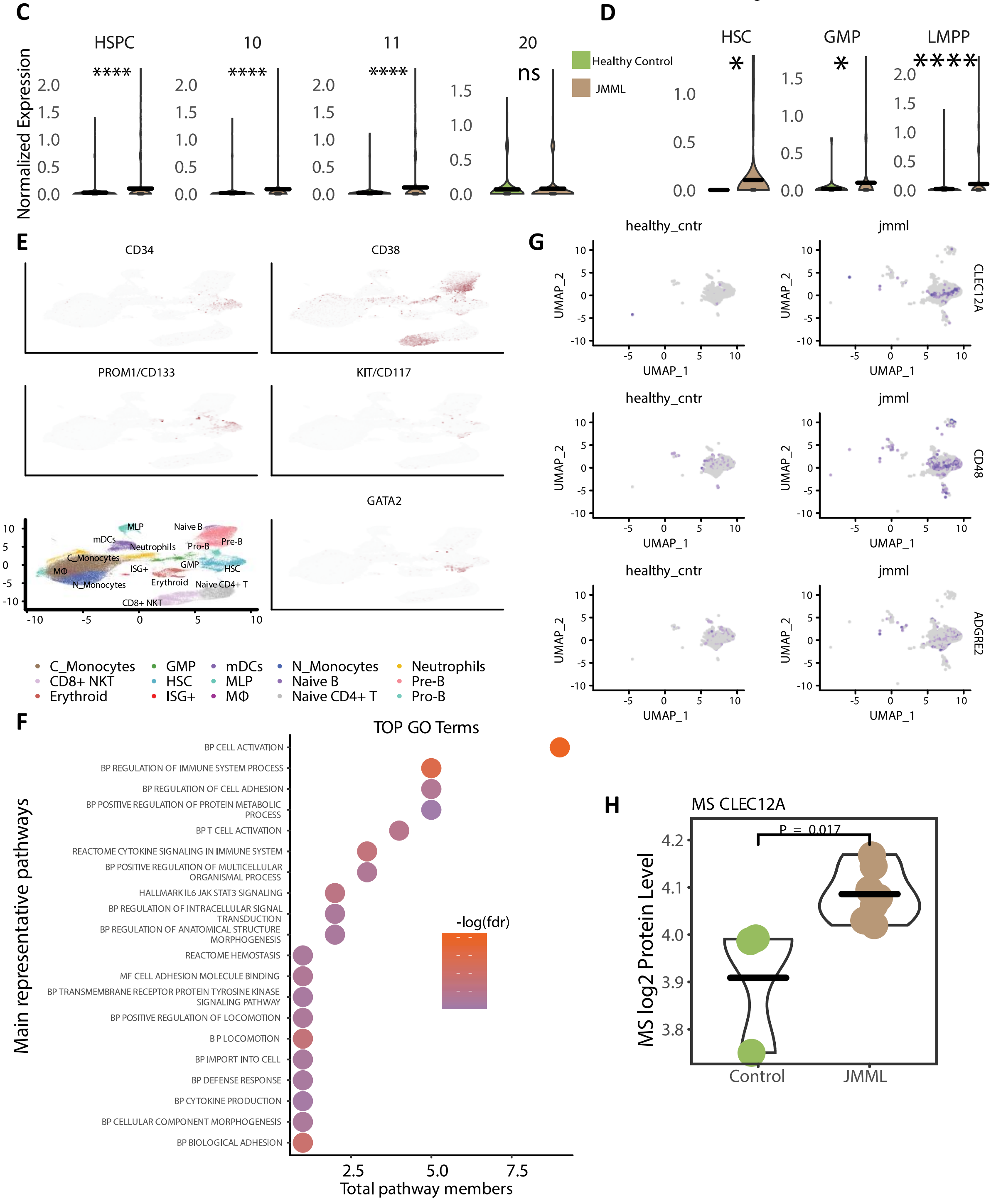

Supplementary Fig. 2: Identification of immunotherapy targets on JMML HSCs by scRNAseq.

(A) To further refine characterization of clusters 10, 11 and 20 within our scRNAseq data, the immunophenotypic markers for HSPCs were referenced (Louka et al., 2021). These marker combinations were adapted these to assess the HSC and HPC populations within our scRNAseq data with the scGate algorithm.

(B) Stacked bar plot representing the relative proportions of HSCs and three types of HSPCs (GMPs, LMPPs, MPPs) in clusters assigned as HSPCs. The "Undetermined" category represents HSPCs that did not show prominent enrichment for specific HSPC markers using scGate analysis.

(C) Violin plots showing the expression of *CLEC12A* in JMML samples compared to healthy controls across clusters HSPC, 10, 11, and 20. Normalized expression is shown on the y-axis.

(D) Violin plots comparing *CLEC12A* expression between JMML and healthy control samples in HSC, GMP, and LMPP populations. Only cell types with a minimum of 100 cells are shown. Normalized expression is on the y-axis. Statistical significance was determined by Bonferroni-corrected Wilcoxon two-tailed test.

(E) UMAPs demonstrating expression of HSC markers in scRNAseq dataset. Bottom plot shows the overall cell type annotations.

(F) Results of GO pathway analysis of upregulated cell surface protein encoding genes: X-axis displays the number of pathway members in each main representative pathway (y-axis) while the dots show the -log10(FDR) for each GO term enrichment.

(G) UMAP depicting expression of the top three CAR T cell targets from combined scRNAseq and mass spectrometry analysis in the respective HSPC cluster.

(H) *CLEC12A* protein levels (log2) measured by mass spectrometry in CD34+ cells. Statistical significance determined using Wilcoxon two-tailed test.

GO=gene ontology, C_Monocytes=classical monocytes, mDCs=myeloid dendritic cells, N_Monocytes=non-classical monocytes, ISG+=ISG expressing immune cells, LMPP= lymphoid-primed multipotent progenitors, MPP=multipotent progenitors, MS=mass spectrometry, Healthy Cntrl=healthy control.

Supplementary Fig. 3

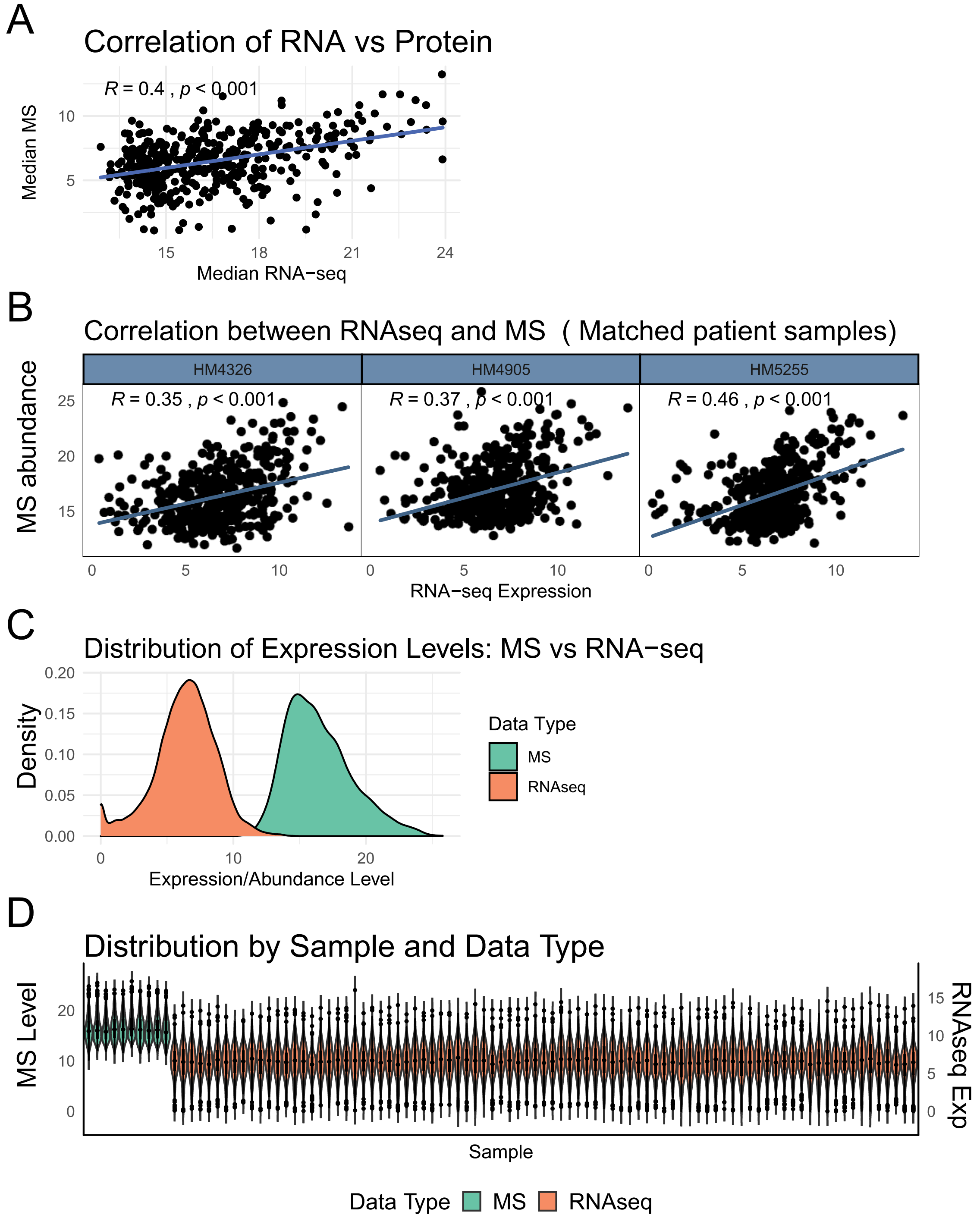

Supplementary Fig. 3: Comparison and correlation of mass spectrometry and bulk RNAseq data.

(A) Correlation between average MS and RNAseq expression levels. Each point represents a gene, plotting its median MS abundance against its median RNAseq expression across all samples. The blue line represents the linear regression fit (Pearson's $r=0.42$, $p<0.001$).

(B) Scatter plots for three patients (HM4326, HM4905, HM5255) between RNAseq and MS expression in matched patient samples. Blue lines show linear regression fits.

(C) Distribution of MS and RNAseq values. The density plot shows the overall distribution of protein abundance (MS, green) and mRNA expression (RNAseq, orange) across all samples.

(D) Distribution of expression levels by sample and data type. Each violin represents a single sample, illustrating the variability within and between samples for both MS (green) and RNAseq (orange) data. Left axis depicts MS protein level while right axis depicts RNAseq expression.

Supplementary Fig. 4

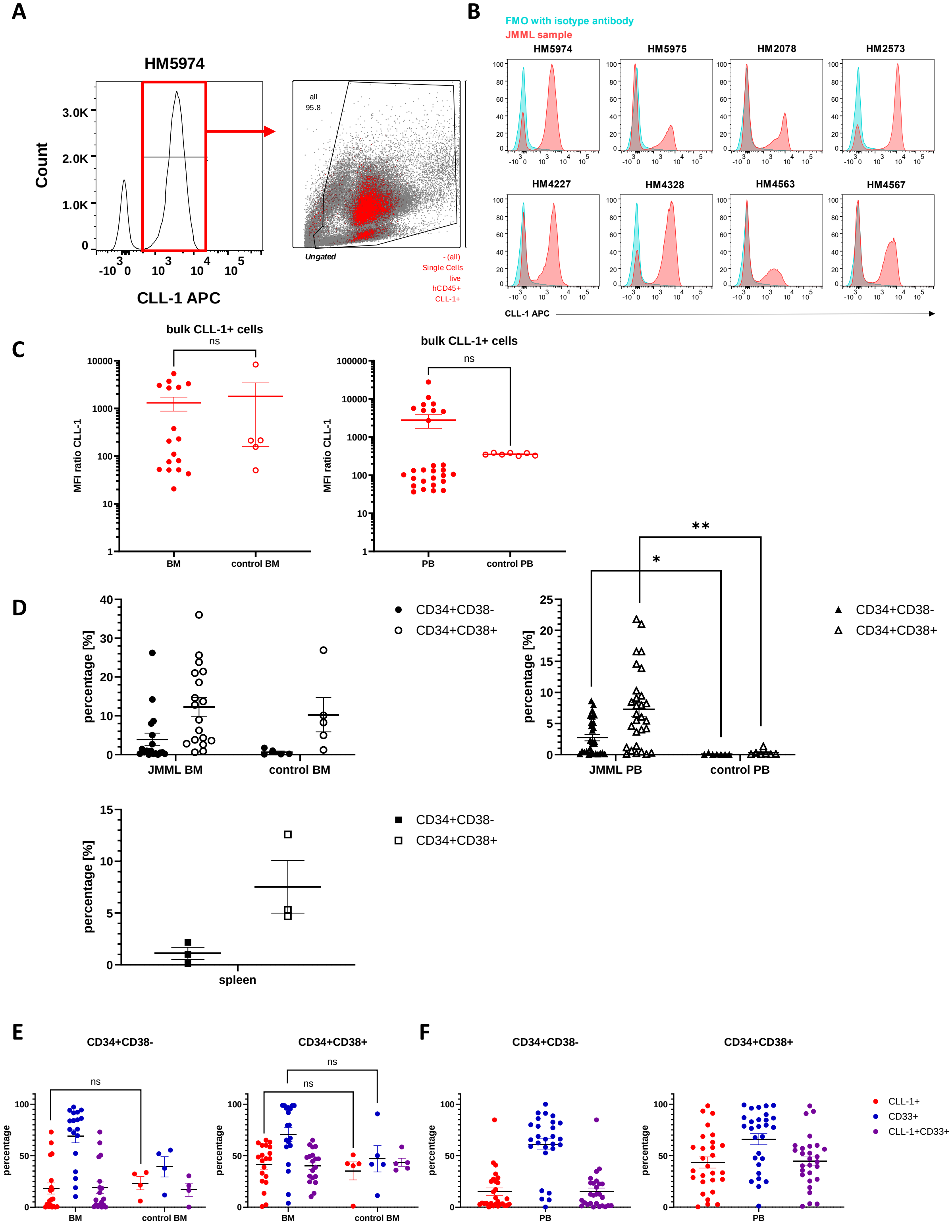

Supplementary Fig. 4: Flow cytometry for CLL-1, CD34 and CD38 on JMML MNCs.

(A) Backgating on CLL-1+ JMML cells up to FSC-SSC gate (representative sample from Extended Data Fig. 3B).

(B) Representative flow cytometry on JMML patient tumor samples from Fig. 3 for CLL-1.

(C) MFI ratio of CLL-1 was assessed for just the CLL-1$^+$ cells from Fig. 3, not all bulk cells. The seemingly bimodal distribution could be due to some samples having MNCs with higher CLL-1 antigen density (also see Fig. 3D).

(D) JMML BM (n=18), healthy donor control BM (n=5), JMML PB (n=29) or healthy donor control PB (n=7) or JMML spleen (n=3) were assessed for percentage of CD34$^+$CD38$^-$ or CD34$^+$CD38$^+$ cells by flow cytometry.

(E) CD34$^+$CD38$^-$ cells from JMML BM (n=19) or healthy donor control BM (n=5), as well as CD34$^+$CD38$^+$ cells from JMML BM (n=20) or healthy donor control BM (n=5) (from Fig. 3E) were assessed for percentage of CLL-1$^+$, CD33$^+$ or double positive cells by flow cytometry. Statistical analysis was performed using an unpaired two-tailed t test.

(F) JMML PB CD34$^+$ populations from Fig. 3E were subset into CD34$^+$CD38$^-$ and CD34$^+$CD38$^+$ cells and assessed for the percentage of CLL-1$^+$ cells in these populations.

Statistical analysis for E and F was performed using unpaired two-tailed t-test (CLL-1) or two-way ANOVA with Šídák's multiple comparisons test (CD33).

Supplementary Fig. 5

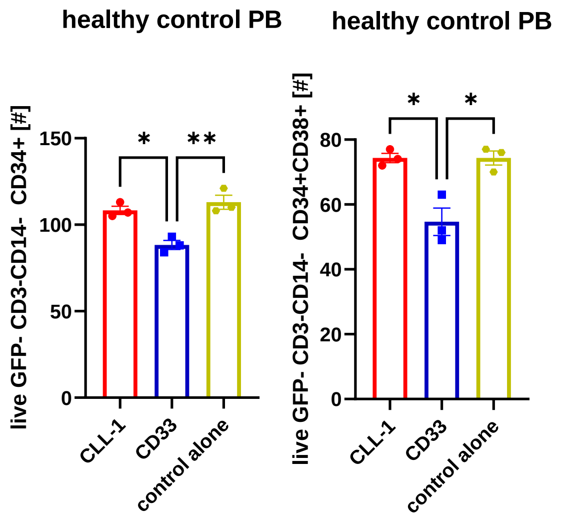

Supplementary Fig. 5: CD33CART reduce healthy HSPCs whereas CLL1CART do not.

CLL1CAR or CD33CART were co-cultured with healthy control PBMCs for 24 hours at E:T ratio 1:1. Remaining live non-T cell $GFP^{-}CD3^{-}$ $CD14^{-}$ $CD34^{+}$ and $CD34^{+}CD38^{+}$ cells (n=3 technical replicates) were assessed by flow cytometry. Due to physiologically very low numbers of $CD34^{+}CD38^{-}$ cells in control PB, this population could not be assessed.

PBMCs=peripheral blood mononuclear cells.

Supplementary Fig. 6

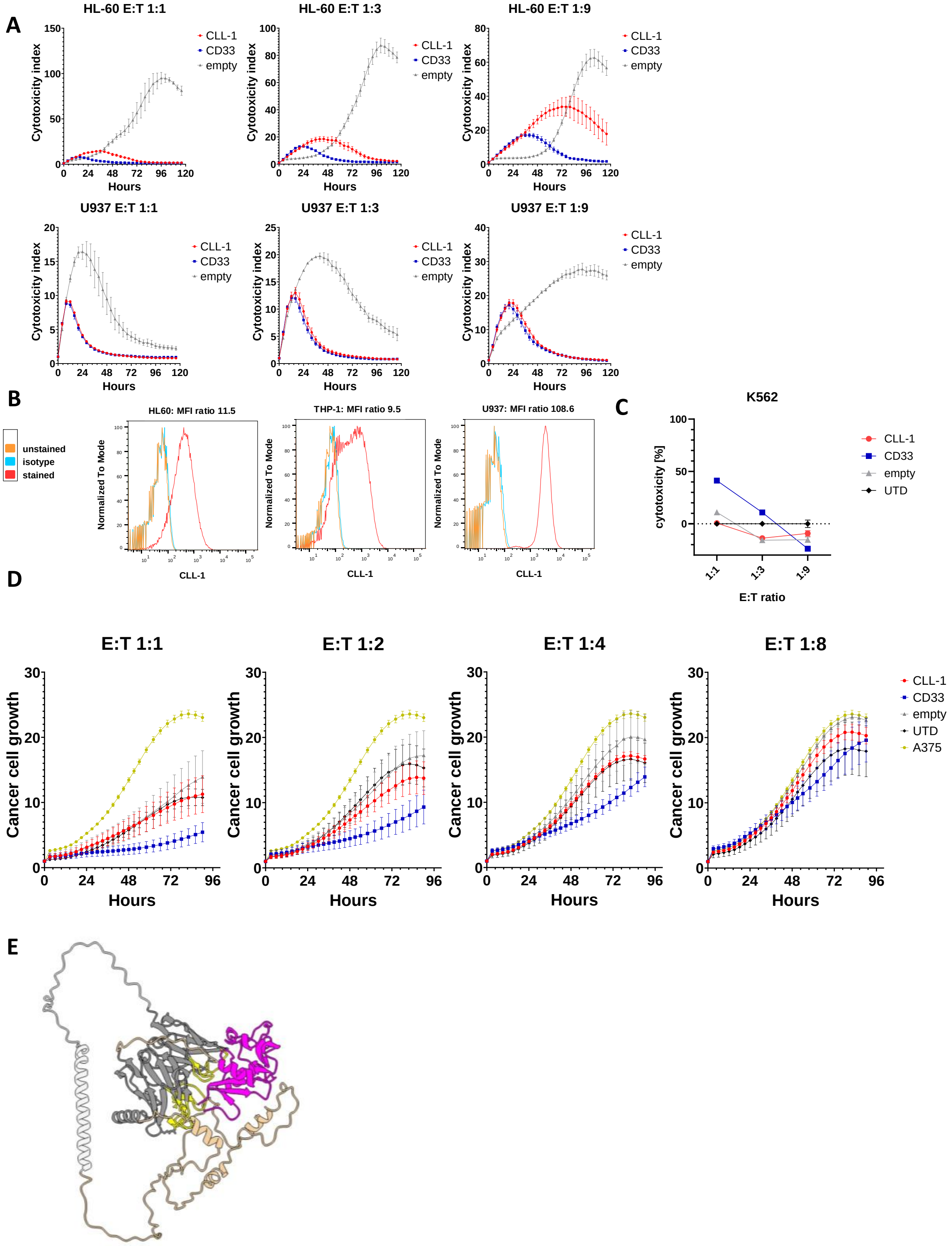

Supplementary Fig. 6: Characterizing function of CLL1CART on CLL-1^{-} and CLL-1^{+} cell lines as well as CLL-1 expression on cell lines.

(A) CLL1CART, CD33CART or empty CAR T cells were co-cultured with mCherry+ CLL-1+/CD33+ tumor cells. Data was obtained using Incucyte live-cell imaging (n=3 technical triplicates). “Total Orange Object Integrated Intensity per well” was normalized to time 0 of the respective well.

(B) CLL-1 expression on HL-60, THP-1 and U937 cell line compared to respective unstained or isotype-stained control cells.

(C) In vitro 24-hour Luciferase-based cytotoxicity assay comparing CLL1CART, CD33CART and empty CAR T cells against K562 cells. Data are normalized to UTD T cells. n=3 technical replicates.

(D) CLL1CART, CD33CART, empty CAR or UTD T cells were co-cultured with A375 melanoma cells. Data was obtained using Incucyte live-cell imaging (n=3 technical triplicates). “Red object count per well” was normalized to time 0 of the respective well.

(E) Predicted 3D structure of the CLL-1 CAR in the “CD8α H+TM 4-1BB backbone”: CLL-1 scFv (active residues 101-256, magenta), anti-CLL-1 M26 antibody clone heavy and light chains (gray), CDRs (yellow), CD8H+TM (light gray) and intracellular domains of CAR (tan).

Supplementary Fig. 7

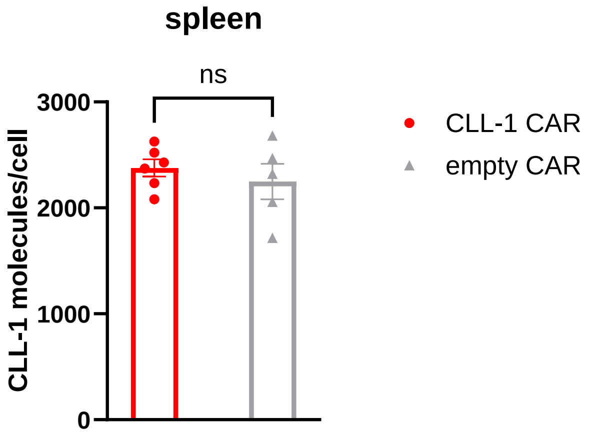

Supplementary Fig. 7: CLL1CART do not induce CLL-1 antigen escape in the U937 in vivo model.

Two weeks after CLL1CART injection, mice were sacrificed and remaining $hCD45^{+}CD3^{-}$ spleen cells were assessed for CLL-1 antigen density. n=6 for CLL1CART, n=5 for empty CAR.

Supplementary Fig. 8

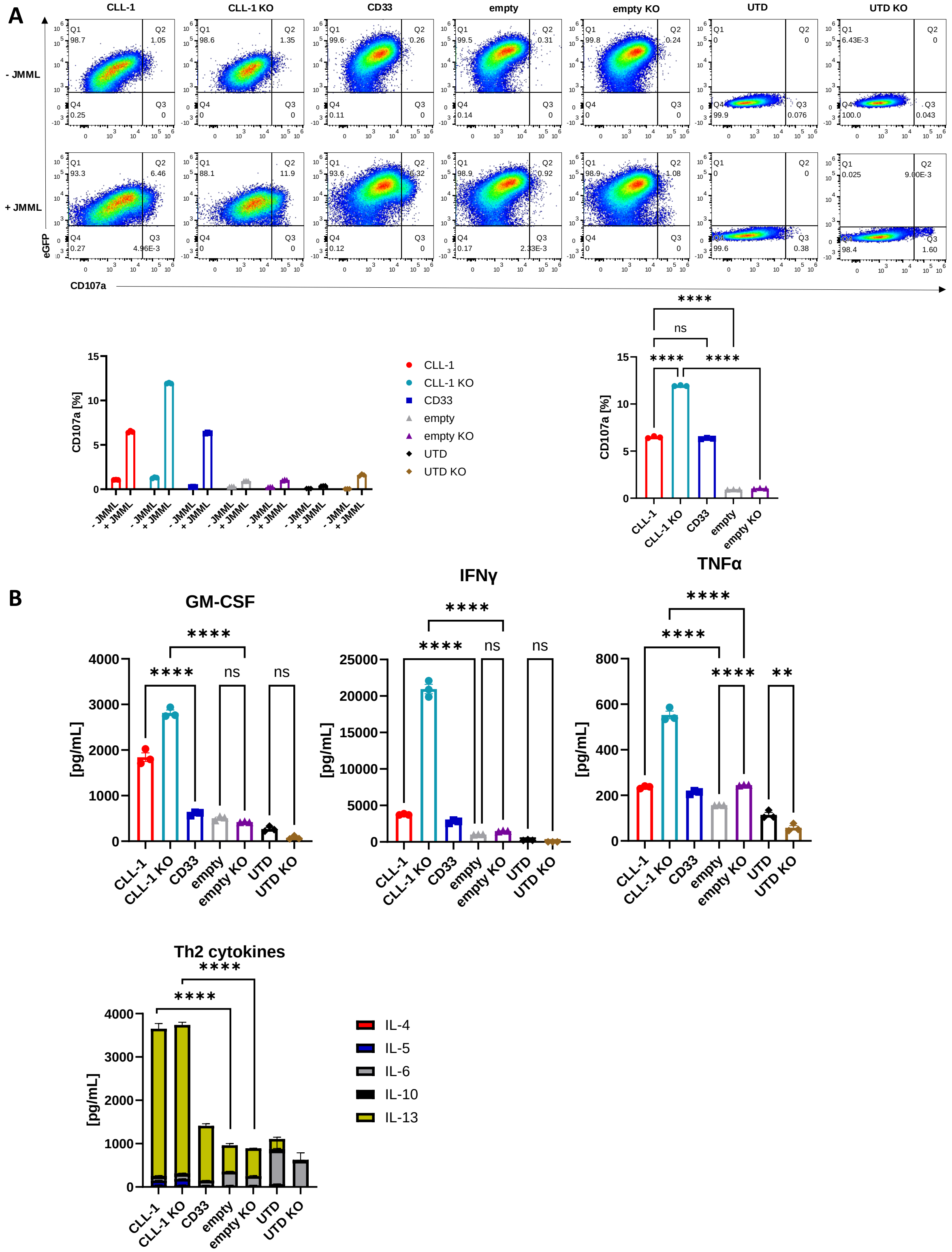

Supplementary Fig. 8: CLL1CAR T degranulate and produce cytokines upon JMML encounter in vitro.

(A) CAR or UTD T cells were co-cultured in the presence or absence of primary JMML cells at a 1:1 E:T ratio for six hours, and then flow cytometry was used to determine degranulation by CD107a expression (n=3 technical triplicates). Statistical analysis was performed by one-way ANOVA with Tukey's multiple comparisons test.

(B) Multiplexed cytokine profiling from culture supernatant after 24 hours of primary JMML exposure (n=3 technical triplicates). For IFNγ a point-to-point semi-log regression was used. For all other cytokines a cubic spline or 5-parameter logistic was applied. One-way ANOVA was performed on individual cytokines. Two-way ANOVA was performed comparing means between treatment groups (main treatment group effect) for Th2 cytokines.

Supplementary Fig. 9

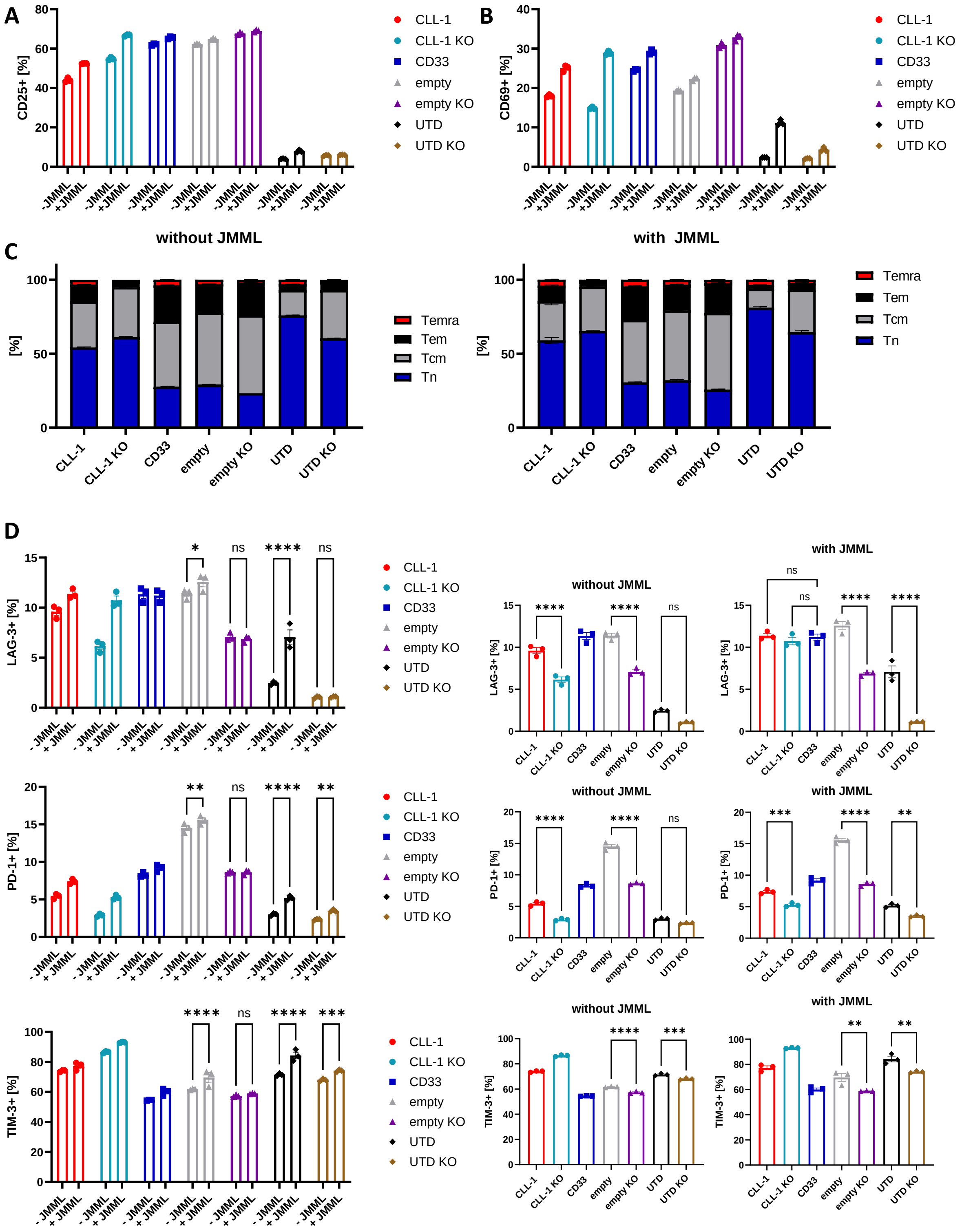

Supplementary Fig. 9: CLL1CART get activated upon JMML exposure in vitro and show similar stemness and exhaustion marker levels than control T cells.

(A/B) CAR or UTD T cells were co-cultured with JMML mononuclear cells from HM5862 at an E:T ratio of 1:1 for 24 hours. Flow cytometry was used to assess activation by staining for CD25 and CD69 (n=3 technical triplicates).

(C) Same setup as in (A/B). Flow cytometry was used to assess stemness markers by staining for CD45RA and CD62L (n=3 technical triplicates).

(D) Same setup as in (A/B). Flow cytometry was used to assess exhaustion markers by staining for LAG-3, PD-1 and TIM-3 (n=3 technical triplicates). Statistical analysis was performed by two- or one-way ANOVA with Tukey's multiple comparisons test.

Supplementary Fig. 10

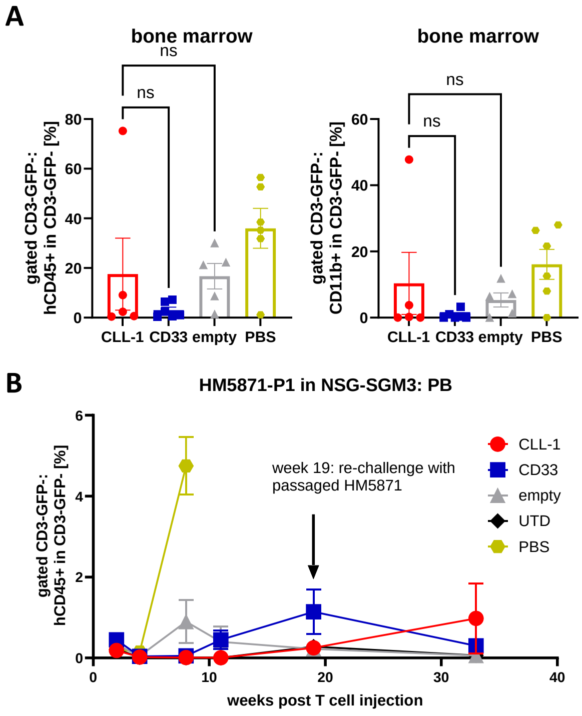

Supplementary Fig. 10: CLL1CART in JMML PDX without *TRAC*-KO shows unspecific cytotoxicity.

(A) Termination data of HM2616 primary sample PDX study in NSG-SGM3 mice. Each mouse received 5e6 CAR T cells (1:1 CD4:CD8 T cells mixed directly before injection). Percentage of engrafted human leukemia or myeloid cells shown. n=5 for CLL-1, empty and PBS; n=6 for CD33. Statistical analysis was performed by one-way ANOVA with Tukey's multiple comparisons test.

(B) Bleeding data for HM5871-P1 PDX study. n=8 mice at study initiation.

P1=passage 1 (harvested twice from spleen of injected mice).

Supplementary Fig. 11

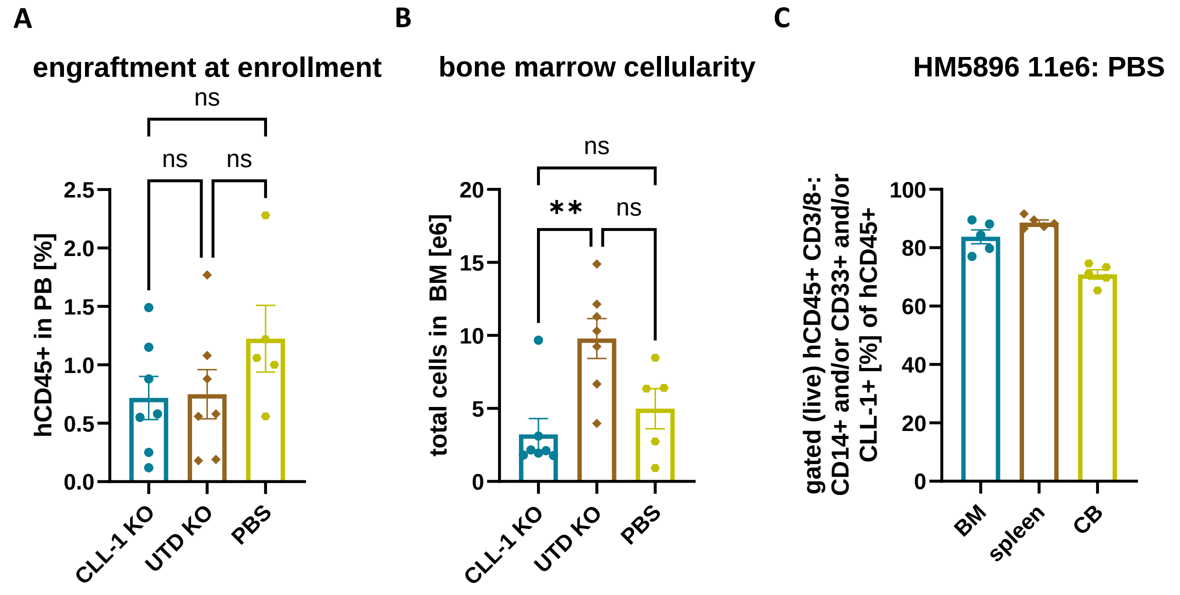

Supplementary Fig. 11: CLL1CART with *TRAC*-KO reduce tumor burden in PB of HM5896 PDX model.

(A) Flow cytometric analysis measuring JMML engraftment in PB at randomization.

(B) Bone marrow cellularity upon termination.

(C) Flow cytometric analysis depicting the percentage of engrafted non-T cell human cells that is assessed by staining for CD14, CD33 and CLL-1 in different tissues.

Statistical analyses were performed by one-way ANOVA with Tukey's multiple comparisons test. (n=7 for CLL-1 KO and UTD KO, n=5 for PBS).

Supplementary Fig. 12

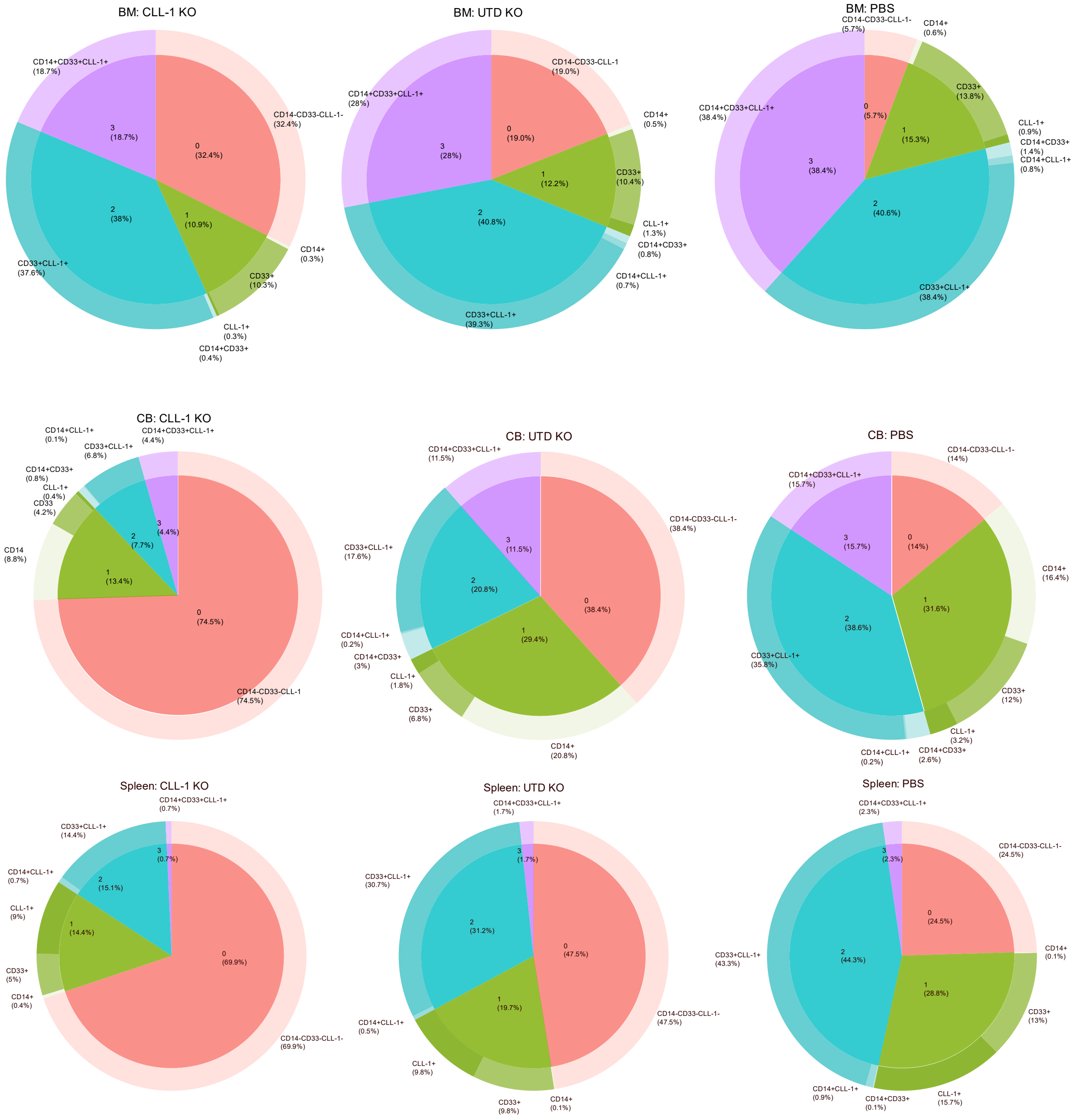

Supplementary Fig. 12: Subsetting JMML burden of the HM2130 termination study

(A) Flow cytometric analysis of BM, spleen and cardiac blood gated on live $hCD45^+$ $CD3/8^-$ cells and assessment of relative percentage of cells that express 0, 1, 2 or 3 markers of CD14, CD33 and CLL-1 within the $hCD45^+$ compartment.

Supplementary Fig. 13

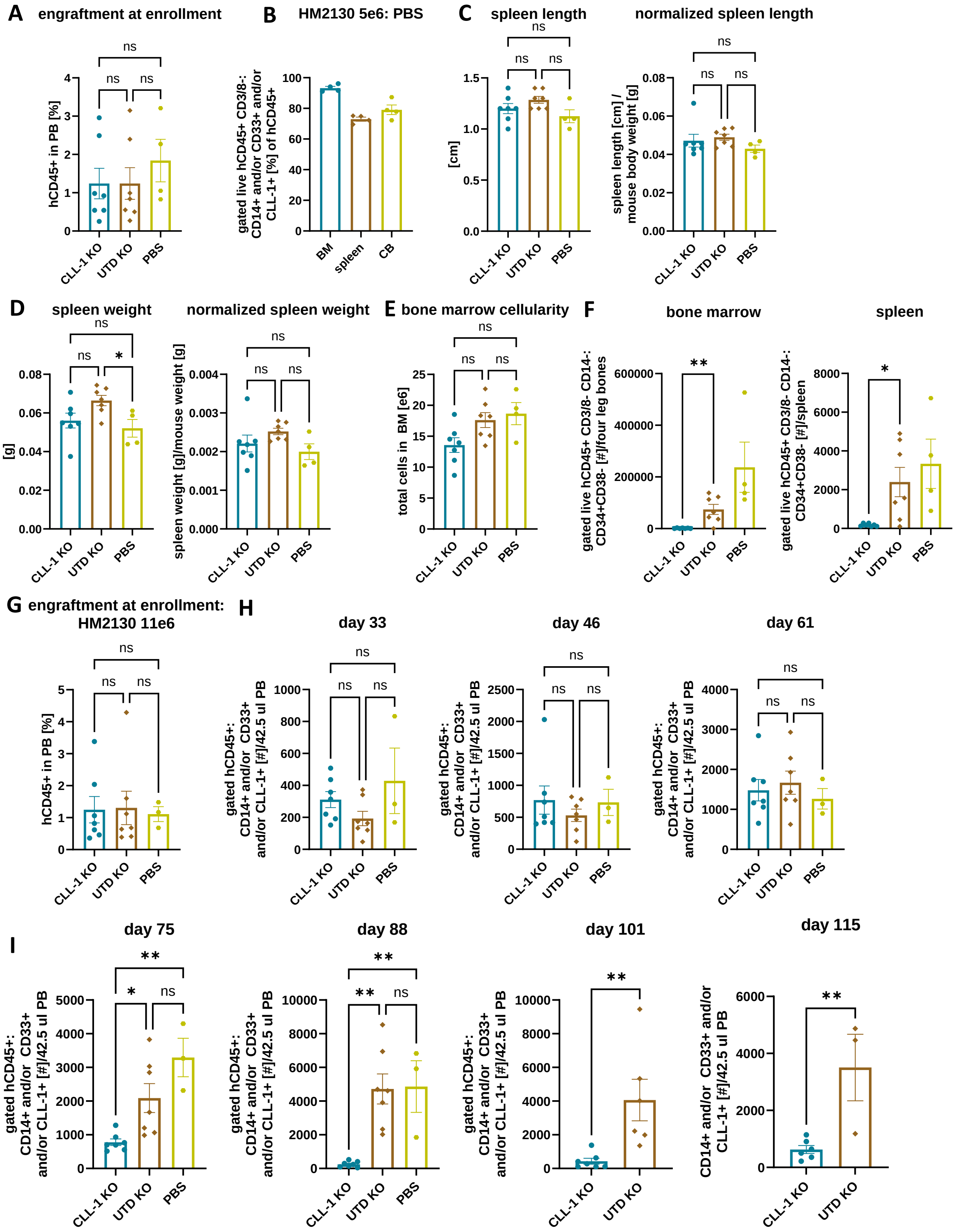

Supplementary Fig. 13: CLL1CART with *TRAC*-KO are effective against HM2130 PDX model.

(A) Flow cytometric analysis measuring JMML engraftment in PB at randomization of the HM2130 termination trial.

(B) Flow cytometric analysis depicting the percentage of $CD3/8^-$ (non-T cell) $hCD45^+$ cells of the HM2130 termination trial that is assessed by staining for CD14, CD33 and CLL-1 in different tissues.

(C) Spleen length and normalized spleen length upon termination of the HM2130 termination trial.

(D) Spleen weight and normalized spleen weight upon termination of the HM2130 termination trial.

(E) Bone marrow cellularity upon termination of the HM2130 termination trial.

(F) Flow cytometric assessment of $CD34^+CD38^-$ cell number in BM and spleen upon termination. Statistical analysis was performed using unpaired two-tailed t-test.

(G) Flow cytometric analysis measuring JMML engraftment of the HM2130 survival trial in PB at randomization.

(H) Flow cytometric analysis over time to assess JMML burden of the HM2130 survival trial in PB before and **(I)** after re-injection of CAR or UTD T cells.

n=7 mice for CLL-1 KO and UTD KO, n=4 for PBS.

Supplementary Fig. 14

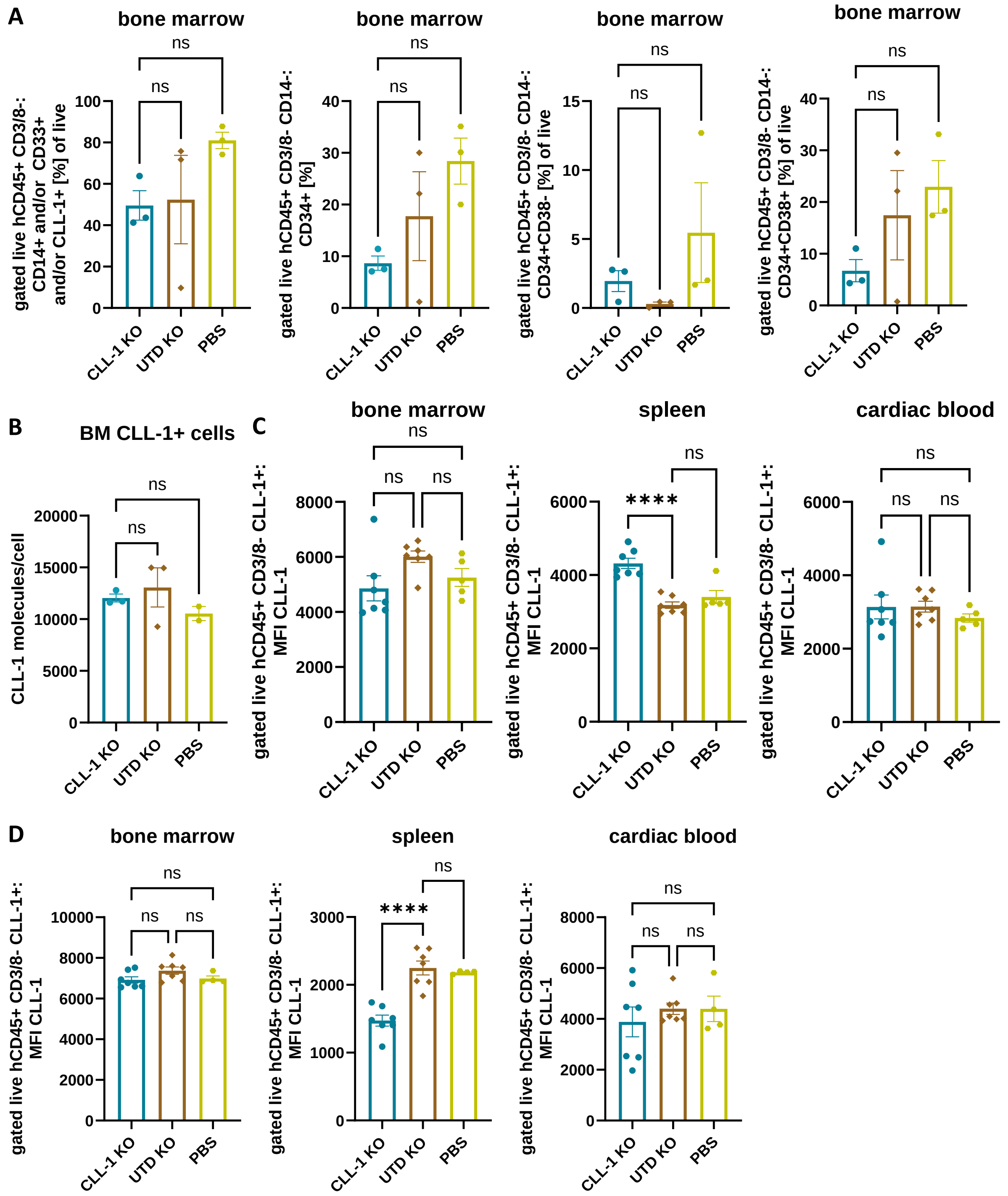

Supplementary Fig. 14: Flow cytometric analysis of the HM2130 survival study, HM5896 and HM2130 termination studies upon humane endpoint.

(A) Flow cytometric analysis of thawed BM vials for JMML and HSPC burden in BM of the HM2130 survival study (n=3 mice/treatment group).

(B) Assessment of CLL-1 by quantification of CLL-1 surface molecules on CLL-1$^+$ cells of BM (same mice as (A)).

(C) Assessment of CLL-1 by MFI of CLL-1 on CLL-1$^+$ cells (HM5896 termination study) (n=7 for CLL-1 KO and UTD KO, n=5 for PBS).

(D) Assessment of CLL-1 by MFI of CLL-1 on CLL-1$^+$ cells (HM2130 termination study) (n=7 for CLL-1 KO and UTD KO, n=5 for PBS).

Supplementary Fig. 15

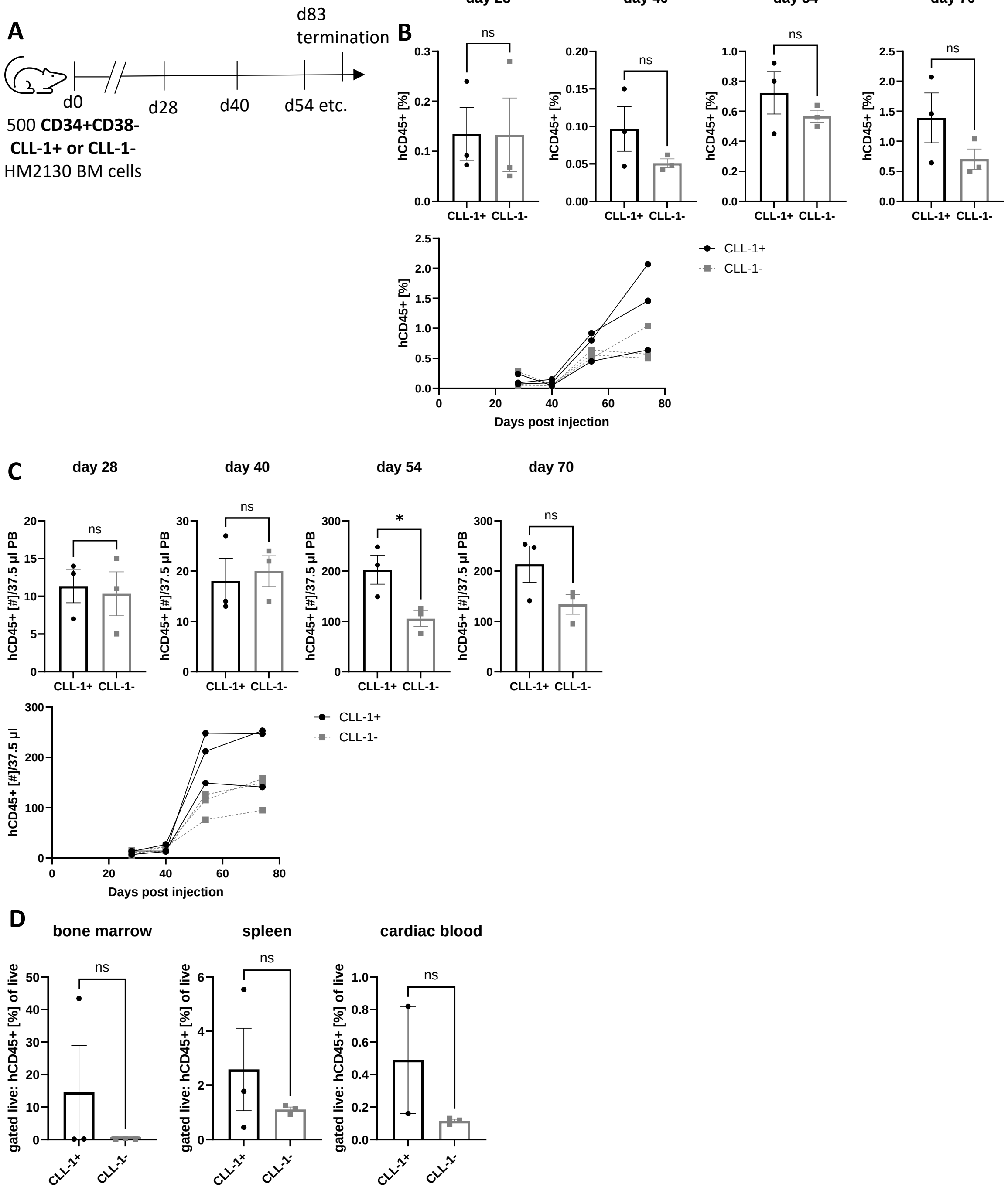

Supplementary Fig. 15: Engraftment potential of JMML CD34⁺CD38⁻ CLL-1⁺ vs. CLL-1⁻ cells.

(A) Schematic for experimental design: Passage 2 HM2130 BM from one NOG-EXL and one NSG mouse were sorted by FACS for CD34⁺CD38⁻ CLL-1⁺ vs. CLL-1⁻ cells and 500 cells/mouse were injected into NOG-EXL mice (n=3/group).

(B) Flow cytometric analysis depicting the percentage of engrafted hCD45⁺ in PB over time.

(C) Flow cytometric analysis depicting the number of engrafted $hCD45^+$ in PB over time.

(D) Flow cytometric analysis depicting the percentage of engrafted $hCD45^+$ JMML cells in end-study tissues.

Supplementary Fig. 16

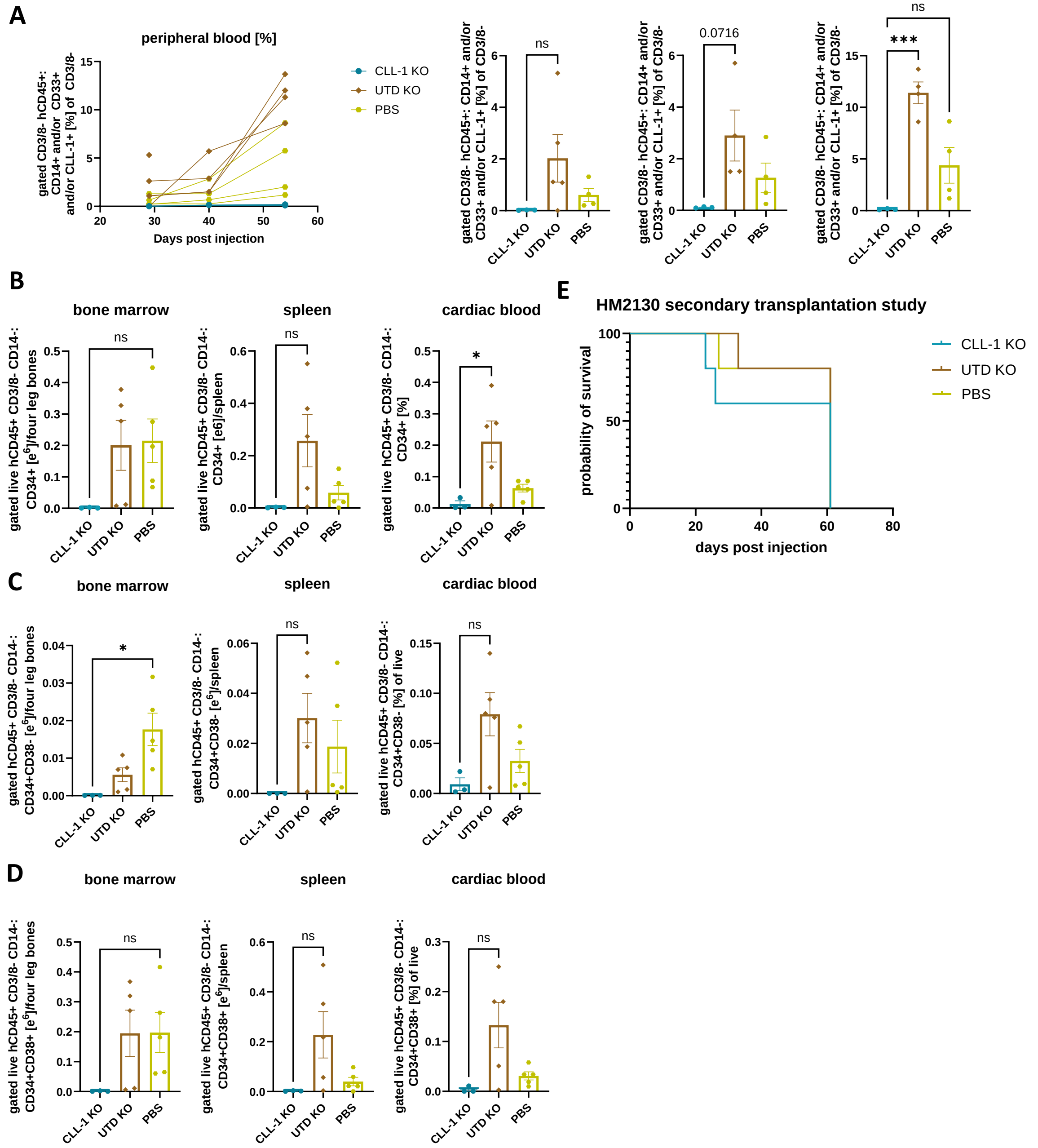

Supplementary Fig. 16: Additional flow cytometric analysis and survival curve of the HM2130 secondary transplantation study.

(A) Flow cytometric assessment of JMML burden [percentage] in PB over time.

(B) Flow cytometric assessment of $CD34^+$ cells in different tissues upon termination using a different unit from Fig. 8.

(C-D) Flow cytometric analysis subsetting cells from (B) into $CD34^+CD38^-$ (C) and $CD34^+CD38^+$ (D) cells.

(E) Survival curve for this study. Two CLL1CART, one UTD and one PBS mouse reached humane endpoint prior to the planned termination on day 61. Their average leukemia burden in BM was 0.5%, 52.5% and 14.8% for the CLL1CART, UTD and PBS mice, respectively. These mice were included into the data presented in Fig. 10 and Extended Data Fig. 10.

Statistical analysis for A-D were performed by one-way ANOVA with Tukey's multiple comparisons test.

n=5 mice/group.

Supplementary Fig. 17

A

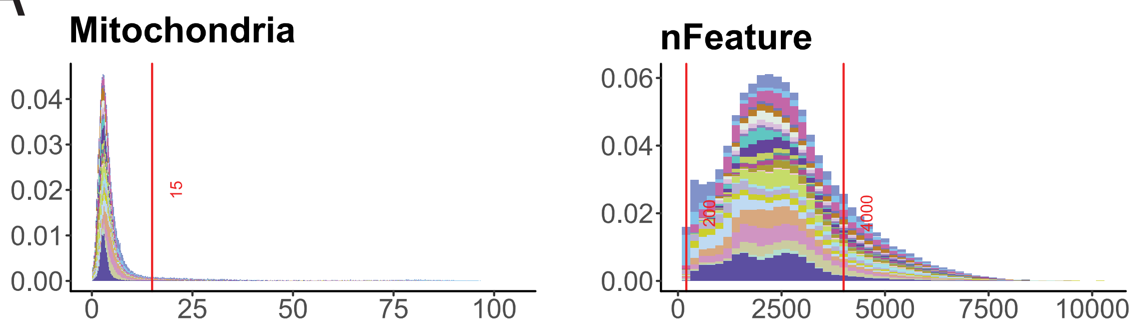

B

sample	nFeature_RNA	nCount_RNA	percent.MT	Total.Before	Total.After
HM5169	754,2840,3999	1112,7375,20381	0.04,2.97,7.5	3467	1733
HM2072	364,2597,3997	1039,7472,28801	0.05,1.51,7.35	2206	1430
HM2616	228,2567,3998	1017,7172,36555	0.05,2.28,8.01	2158	1453
HM4566	321,2616,3996	1266,7822,24463	0.02,2.07,7.18	1220	668
HM2494	281,2181,3997	1021,6088,20154	0.08,3.19,6.7	3302	2656
HM2463	266,2180,3996	1070,6024,18974	0.15,2.4,5.85	3236	3039
HM2159	470,2354,3999	1001,7037,20204	0.09,2.58,6.22	4559	3234
HM4106	249,2789,3999	1020,7497,22301	0.03,1.61,5.91	3272	2512
HM3294	286,2244,3996	1108,7179,40895	0.14,2.97,6.88	5196	4643
HM3679	243,2092,3968	1046,6074,19963	0.02,3.24,7.75	2227	2184
HM3158	218,1915,3980	1012,5270,19719	0,2.38,6.53	3630	3463
HM4924	203,2414,3999	1004,7849,22407	0,2.48,7.04	6677	6295
HM4210	315,2668,3992	1065,8984,25770	0,1.49,7.77	3964	2907
HM4155	258,3050,3998	1045,9271,23177	0.04,2.55,8.9	1372	603
HM4085	630,2706,3998	1285,8928,25071	0,2.81,6.94	1337	765
HM5746	202,2332,3993	1122,6609,28948	0,2.46,6.32	1862	1576
HM5230	202,1961,3999	1013,7442,37399	0,4.34,15	5957	4188
HM5421	473,2375,3998	1002,8066,36287	0.03,3.67,14.98	5121	3732
HM5436	241,1566,3998	1001,3770,33723	0,2.85,14.96	7803	6322
HM5446	303,2252,3998	1005,7264,24131	0,4.62,15	9889	7778
HM5550	385,2301,3999	1004,7235,26651	0,5.02,14.99	10038	8008
HM5698	356,2748,3999	1011,8306,32703	0.05,5.05,14.99	6982	5473
HM5810	206,2180,3999	1001,6322,20224	0,3.02,14.98	15480	13596
HD_2117	253,2564,3999	1006,6942,42681	0,6.67,14.99	11021	4644
HD_2471	505,3070,3999	1003,10043,34757	0.26,7.42,14.99	6878	2086
HD_2689	307,2549,3999	1005,7196,42749	0,4.34,15	11529	7799

* Final: Min, Median, Max

Supplementary Fig. 17: Quality control (QC) metrics for scRNAseq.

(A) Histogram distributions of quality control metrics for all samples. Each sample is represented by a different color and plotted together. Red dotted lines denote QC thresholds used for filtering. The left panel shows the percentage of mitochondrial genes expressed per cell, while the right panel displays the distribution of the number of detected genes (nFeature_RNA) per cell.

(B) Table summarizing the final attributes of all samples after quality control filtering. The white columns show the three features used for QC filtering: percentage of mitochondrial genes (percent_MT), number of detected genes (nFeature_RNA), and number of counted RNA molecules (nCount_RNA). Numbers represent the minimum, median, and maximum values for each of these attributes in the final dataset. The last two columns indicate the total cell count before and after QC filtering for each sample.

Supplementary Fig. 18

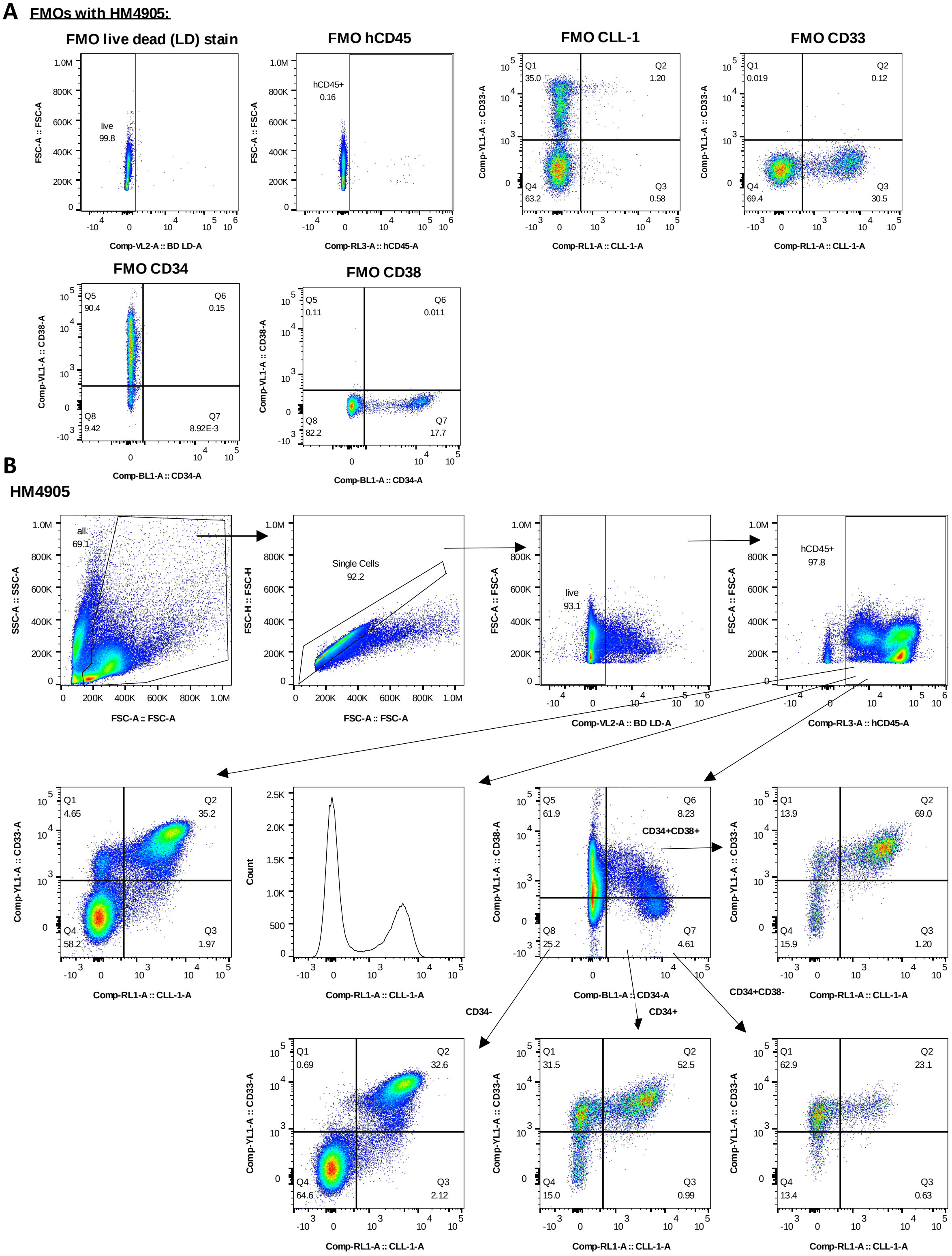

Supplementary Fig. 18: Representative flow cytometry gating plots.

(A) Assessment of CLL-1, CD33, CD34 and CD38 expression on JMML bulk cells as well as different HSPC populations (Fig. 3 and Supplementary Fig. 4).

Supplementary Fig. 19

HM2616

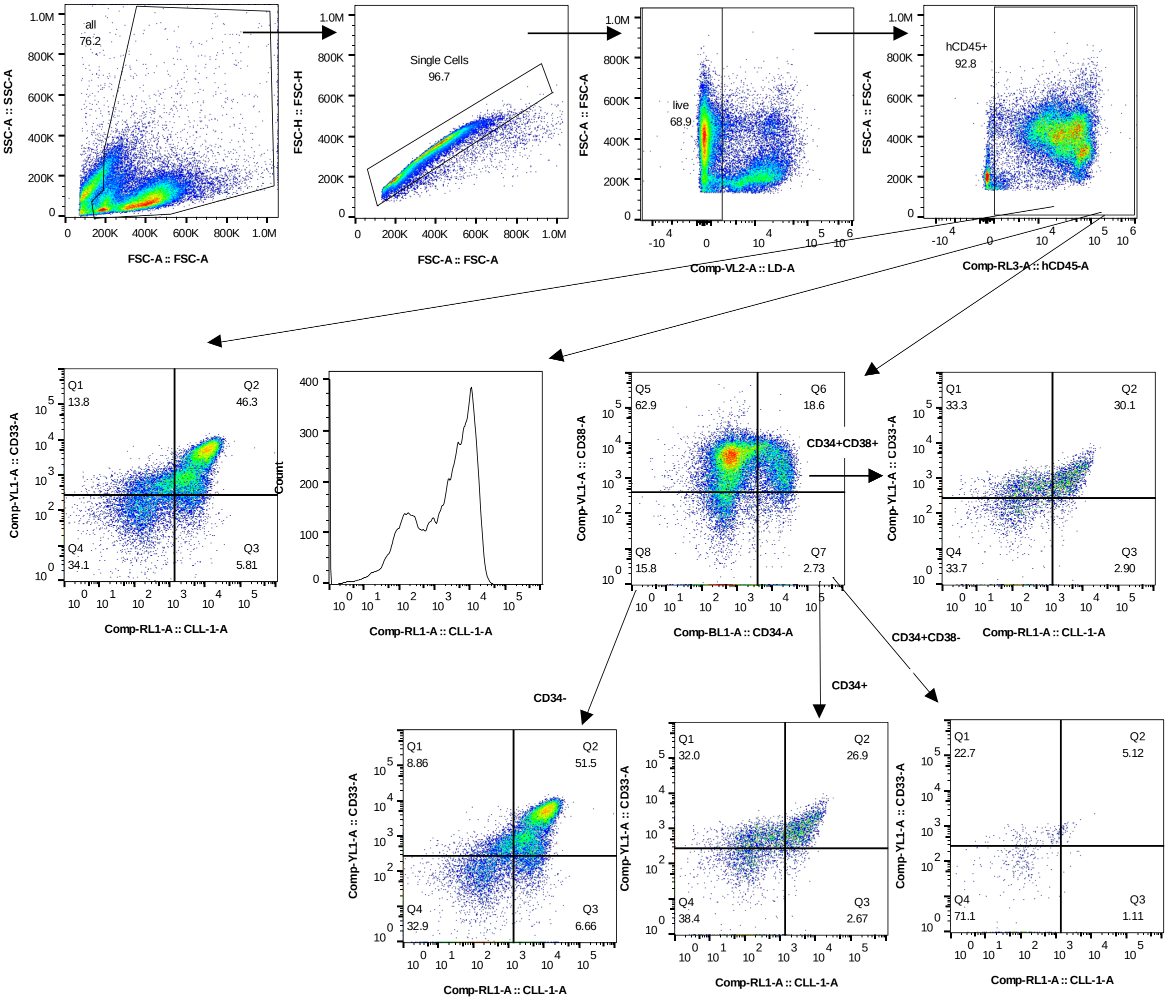

Supplementary Fig. 19: Additional JMML sample illustrating gating strategy from Supplementary Fig. 18 – HM2616 (Fig. 4E)

Supplementary Fig. 20

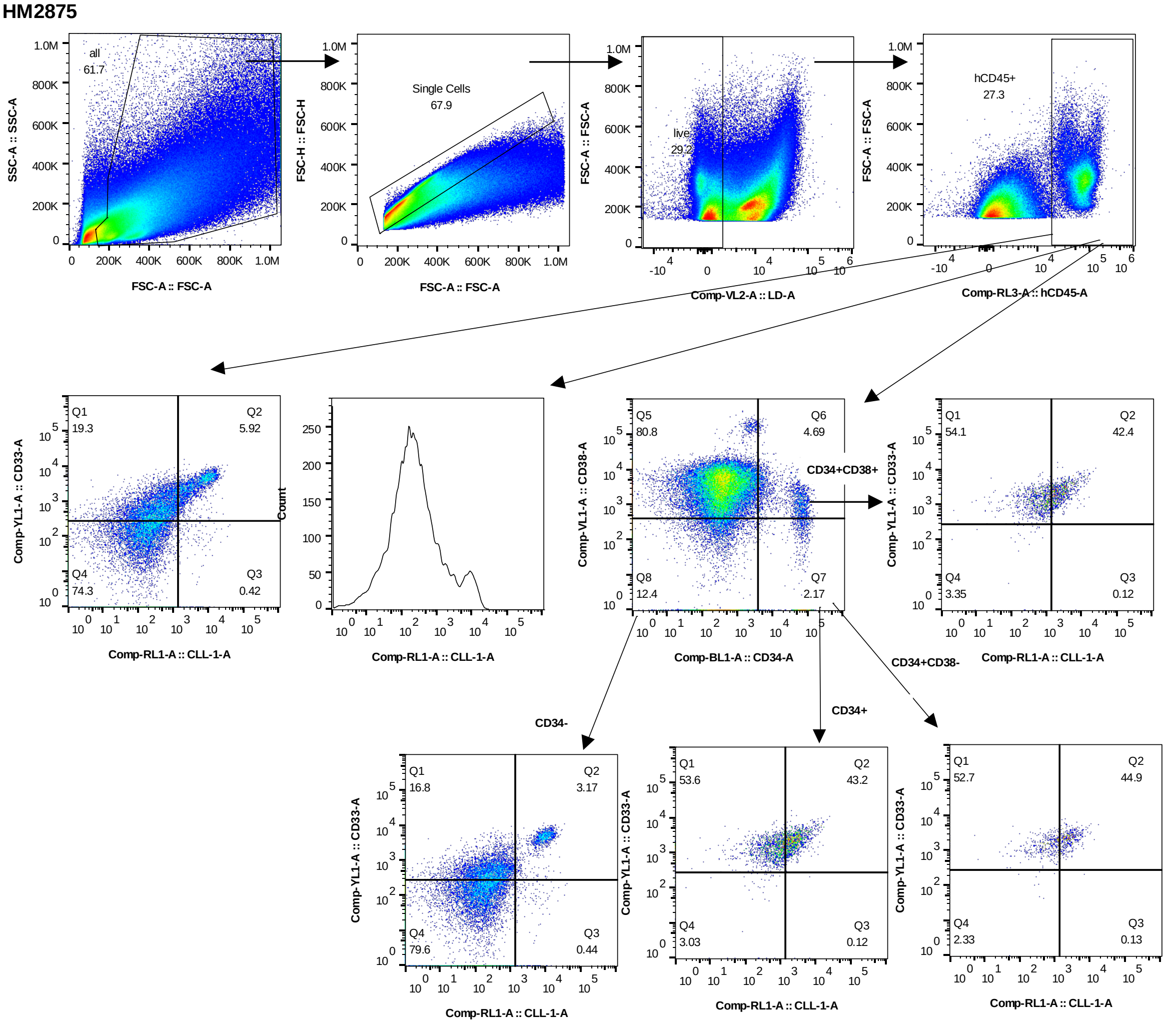

Supplementary Fig. 20: Additional JMML sample illustrating gating strategy from Supplementary Fig. 18 – HM2875

Supplementary Fig. 21

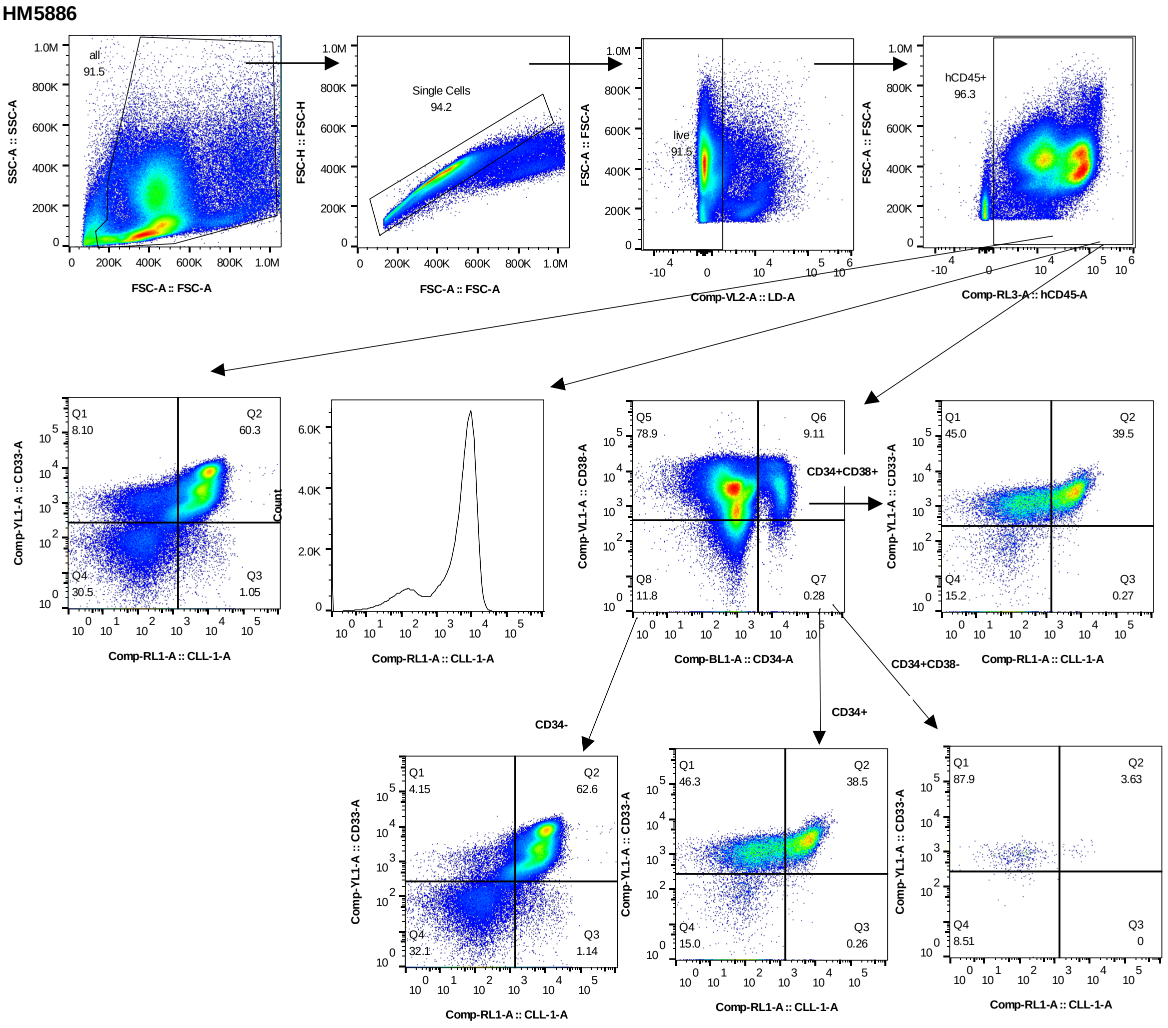

Supplementary Fig. 21: Additional JMML sample illustrating gating strategy from Supplementary Fig. 18 – HM5886

Supplementary Fig. 22

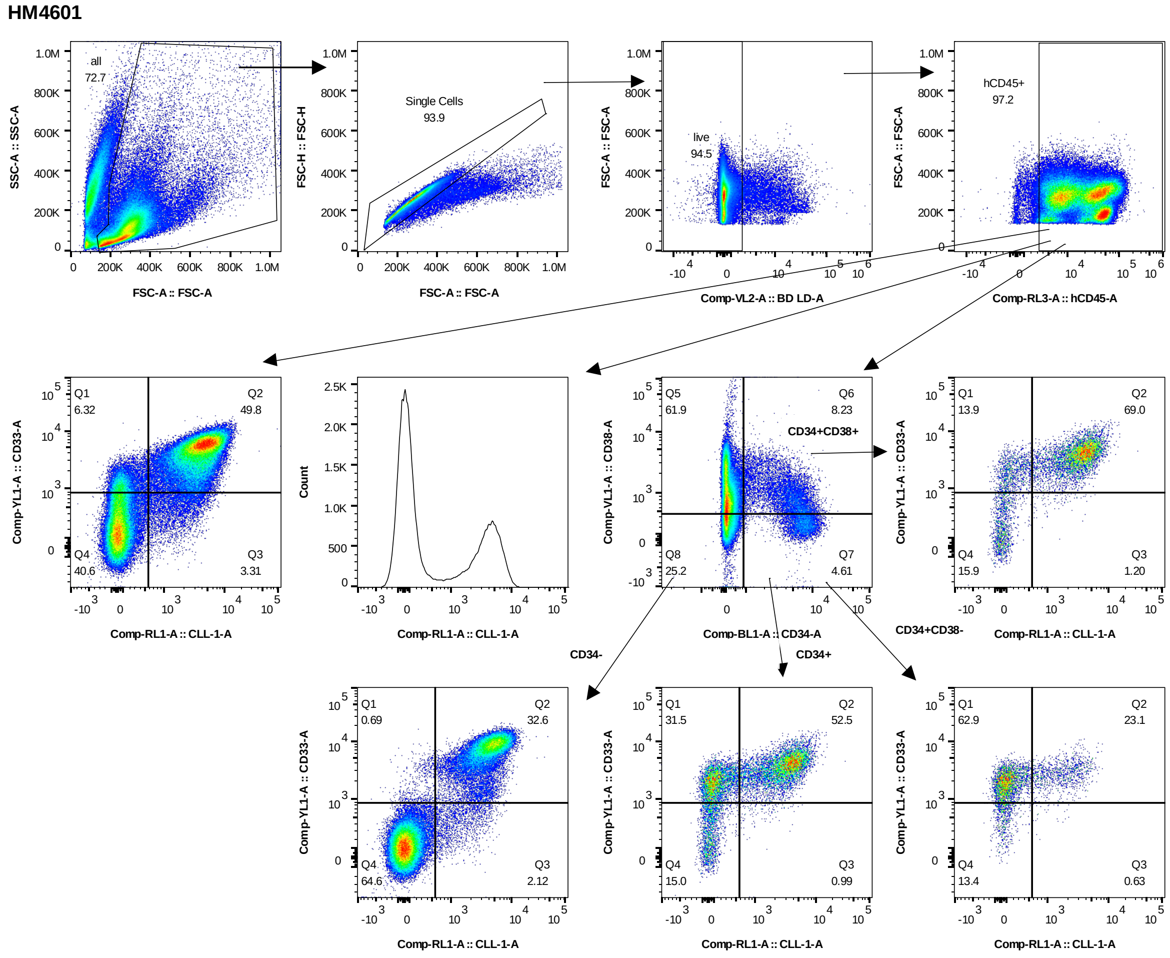

Supplementary Fig. 22: Additional JMML sample illustrating gating strategy from Supplementary Fig. 18 – HM4601

Supplementary Fig. 23

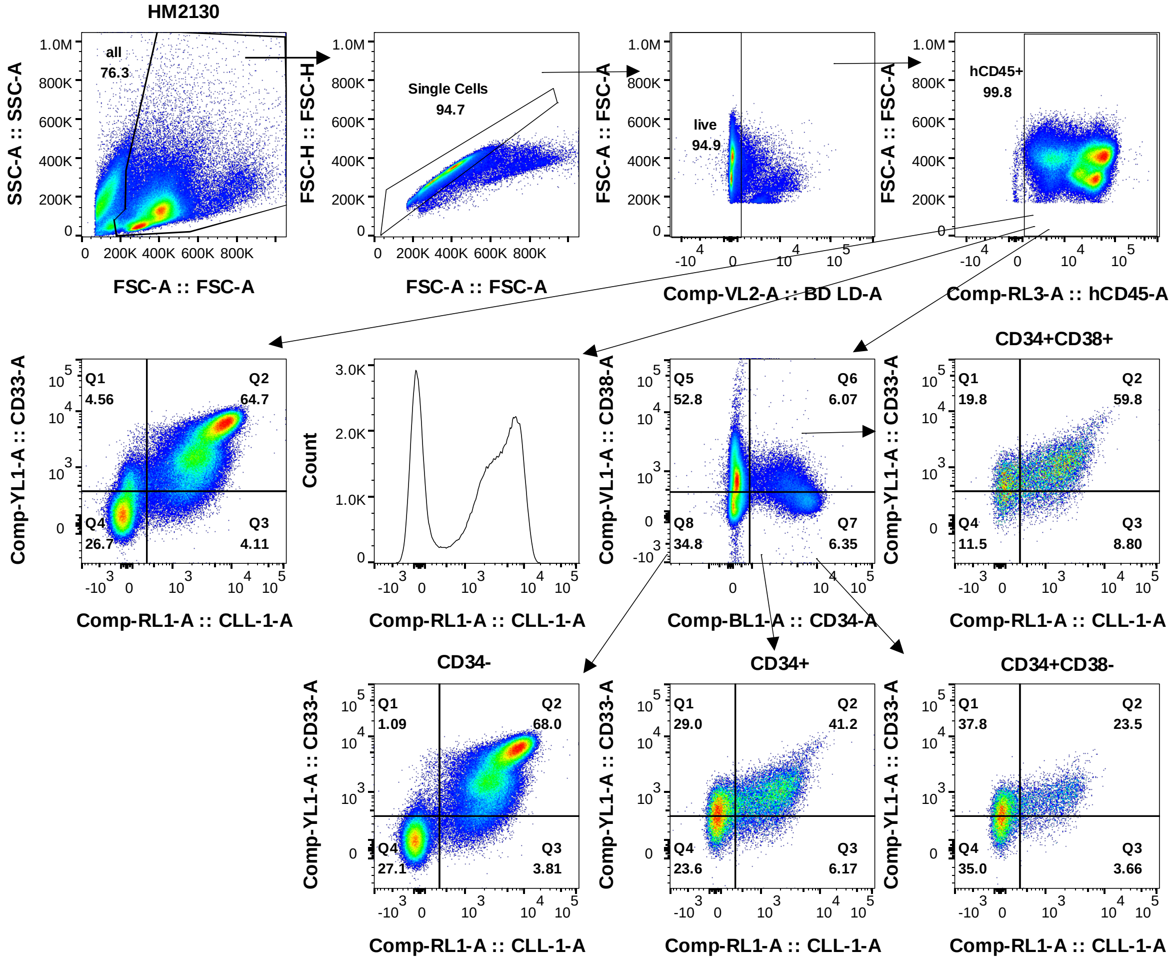

Supplementary Fig. 23: Additional JMML sample illustrating gating strategy from Supplementary Fig. 18 – HM2130 (Fig. 7-8, Supplementary Fig. 14, 16)

Supplementary Fig. 24

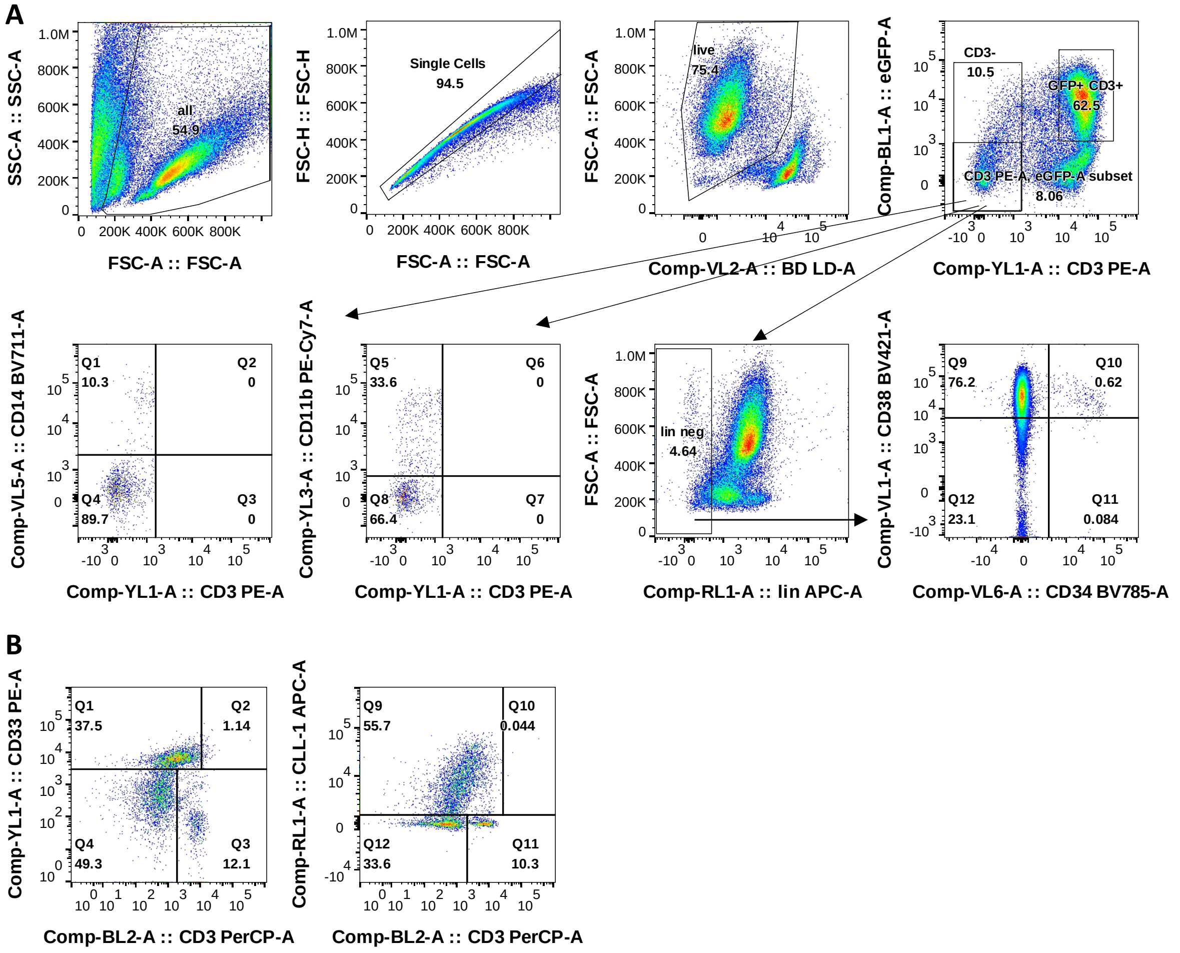

Supplementary Fig. 24: Flow cytometry gating strategies.

(A) For Fig. 4C-D, F-G.

(B) For Fig. 4E (rest of gating strategy similar to (A): FSC-A vs. SSC-A → FSC-A vs. FSC-H → live → GFP$^-$ → depicted plots).

Supplementary Fig. 25

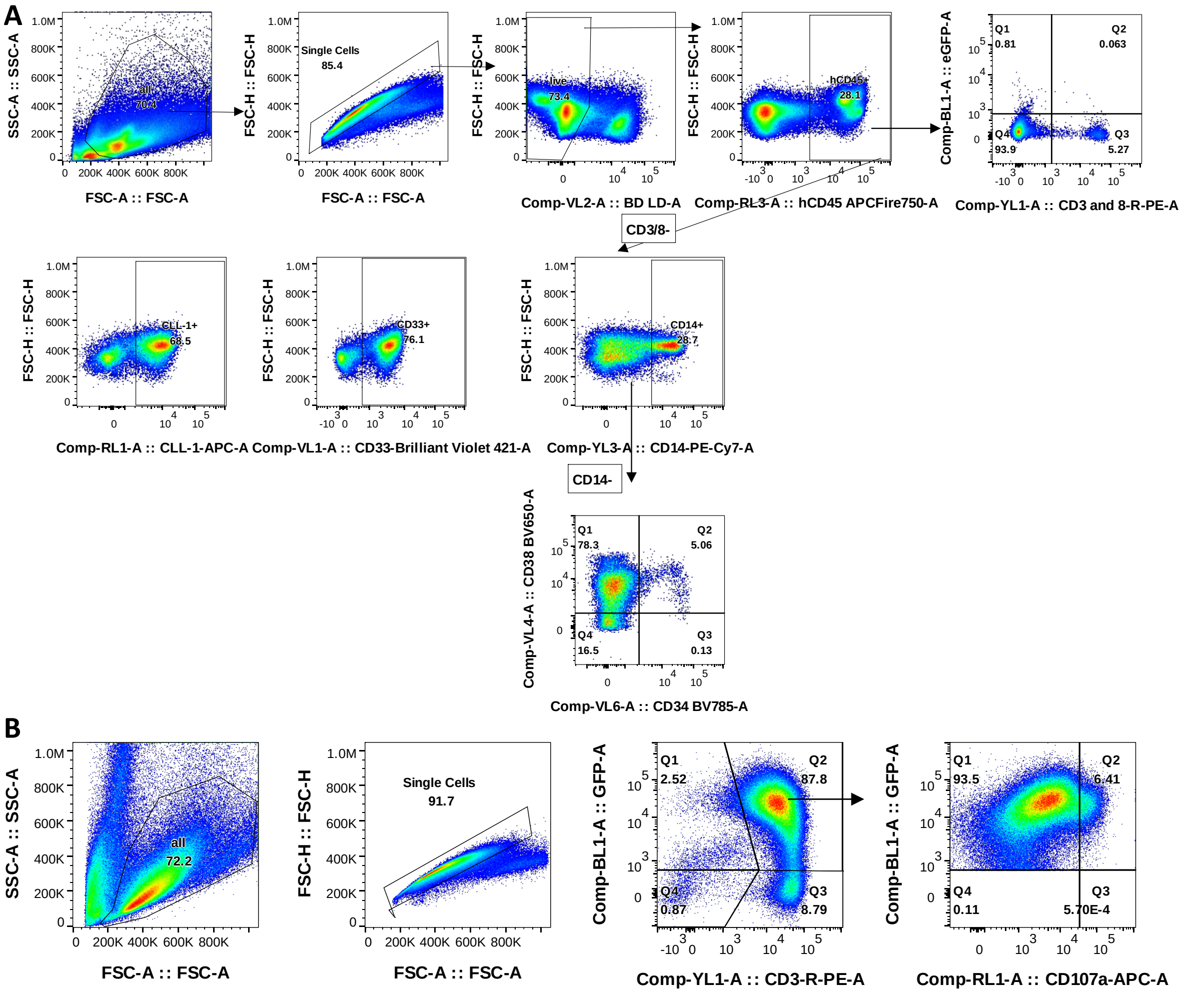

Supplementary Fig. 25: Flow cytometry gating strategies.

(A) Assessment of tumor and HSPC burden in the termination and survival studies (e.g., Fig. 6B, 7B, 7H, Supplementary Fig. 13G-H (without live dead stain/gate); Fig. 6C-F, 7C-F, Supplementary Fig. 11C/13B.

(B) For Supplementary Fig. 8A (respective CD3 vs. eGFP quadrant was chosen based on treatment group prior to CD107a gate: UTD → CD3^{+} GFP^{-}, UTD KO → CD3^{-} GFP^{-}, CAR →CD3^{+} GFP^{+}, CAR KO → CD3^{-} GFP^{+})

Supplementary Fig. 26

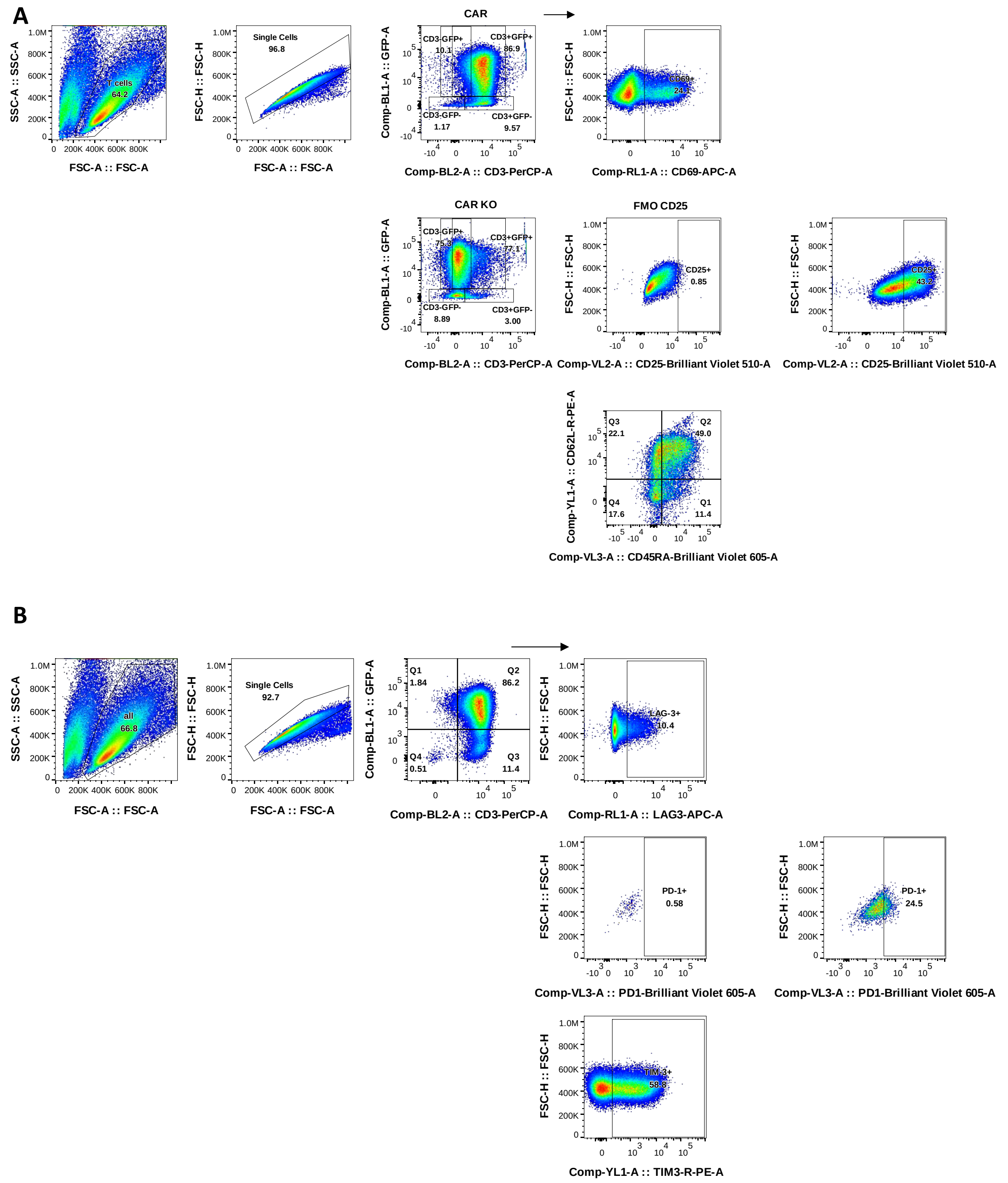

Supplementary Fig. 26: Flow cytometry gating strategies.

(A) For Supplementary Fig. 9A-C (respective CD3 vs. eGFP quadrant was chosen based on treatment group prior to CD25/CD69/CD45RA vs. CD62L gate: UTD → $CD3^+$ GFP^-, UTD KO → $CD3^-$ GFP^-, CAR/CAR KO → GFP^+)

(B) For Supplementary Fig. 9D (respective CD3 vs. eGFP quadrant was chosen based on treatment group prior to LAG3/PD1/TIM3 gate: UTD → $CD3^+$ GFP^-, UTD KO → $CD3^-$ GFP^-, CAR →$CD3^+$ GFP^+, CAR KO → $CD3^-$ GFP^+).

Supplementary Fig. 27

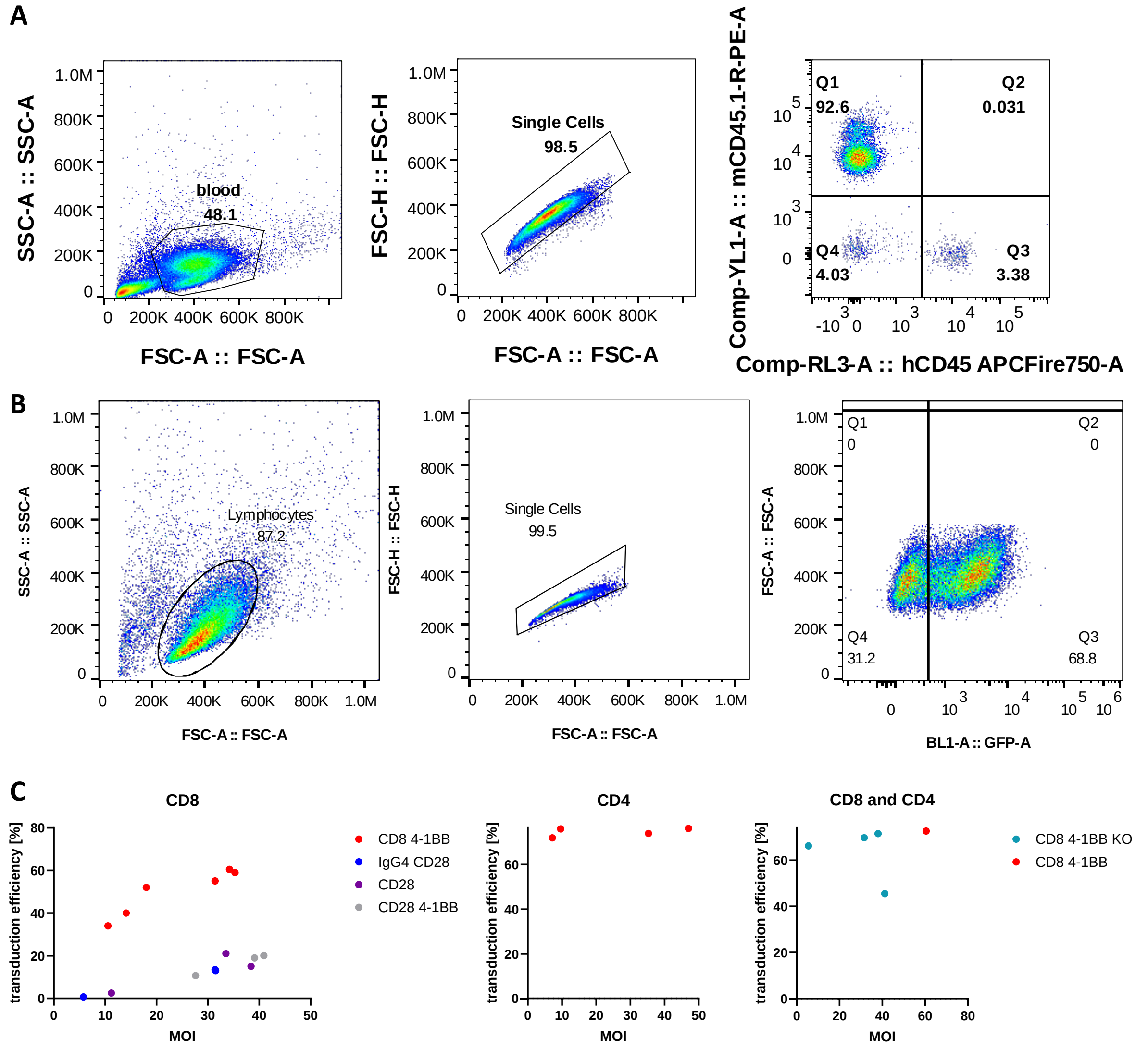

Supplementary Fig. 27: Flow cytometry gating strategies.

(A) For SupplementaryFig. 11A/13A/13F.

(B) Assessment of transduction efficiencies of CAR vectors into T cells by eGFP expression.

(C) Flow cytometric analysis of eGFP expression as marker of CLL-1 CAR construct transduction efficiency of CD8, CD4 or pooled (after thawing) CD8 and CD4 T cells. Analysis was performed between 5-11 days after transduction. Multiplicity of infection (MOI) was calculated based on cell count after thawing two days before transduction.

Supplementary Data Fig. 28

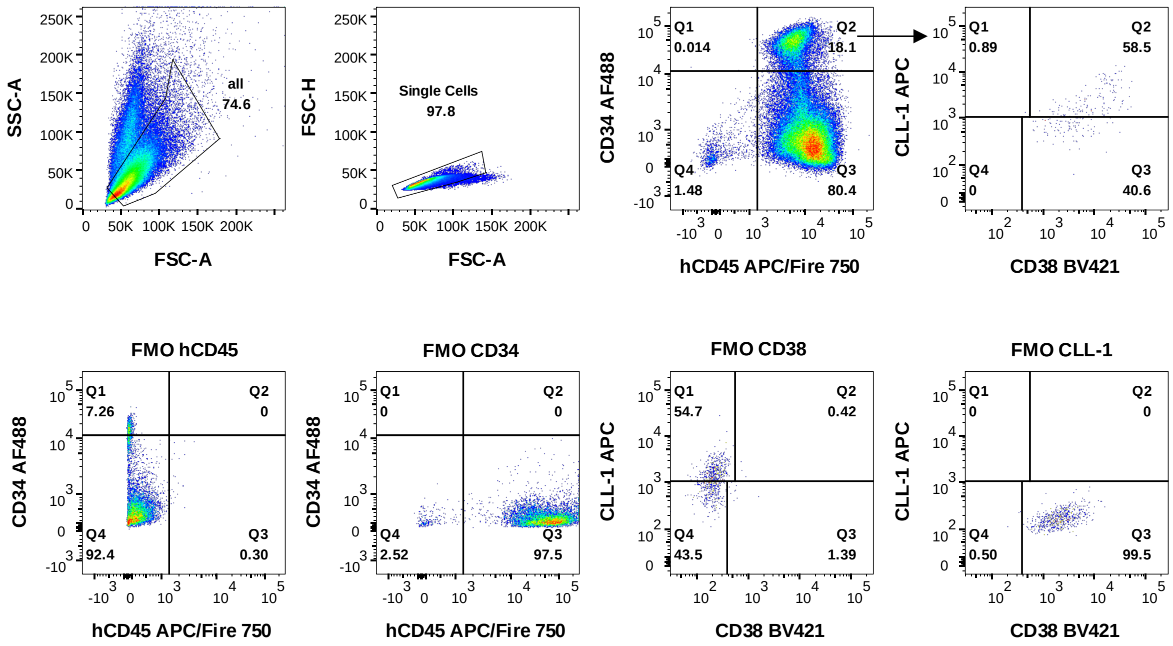

Supplementary Fig. 28: Flow cytometry gating strategy (upper row) for injection of CLL-1$^+$ vs. CLL-1$^-$ CD34$^+$CD38$^-$ cells into mice (lower row: FMOs) (Supplementary Fig. 15).

Supplementary Fig. 29

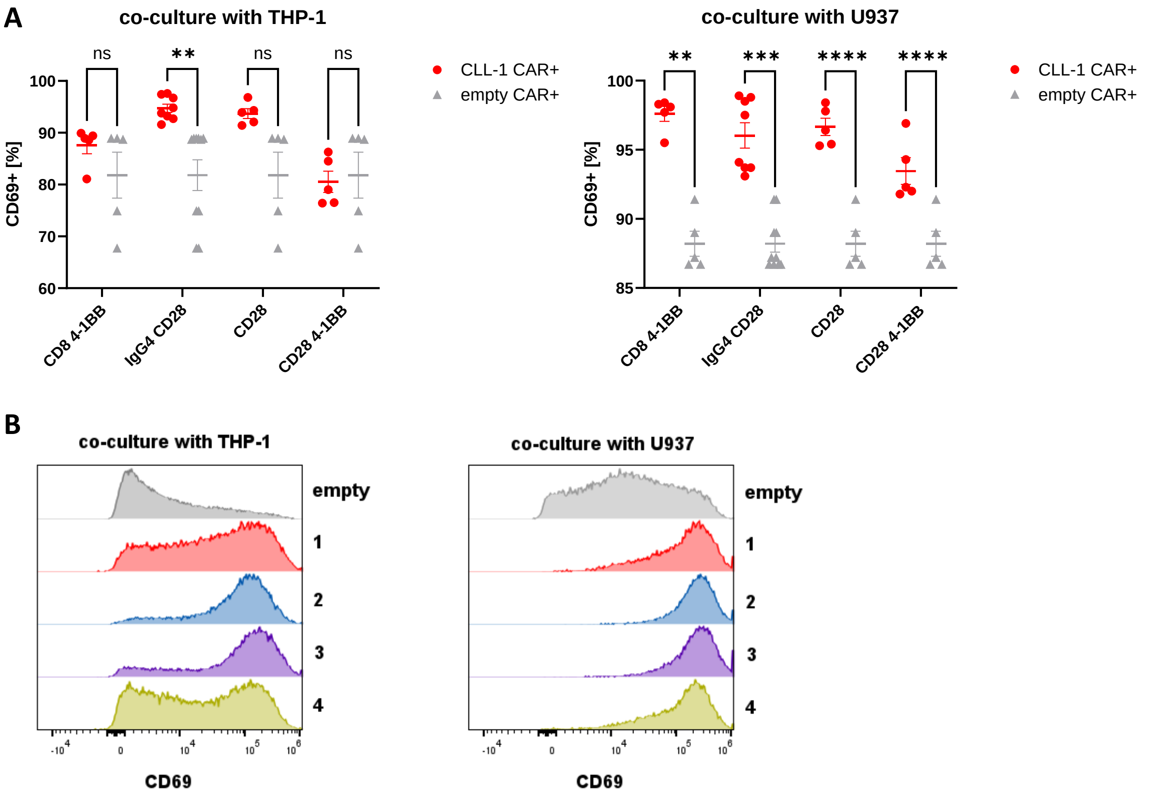

Supplementary Fig. 29: CLL-1 CAR expressing Jurkat cells get activated upon CLL-1 exposure.

(A) The CLL-1 or empty CAR was transduced into Jurkat T cells. These cells were exposed to their CLL-1 target on THP-1 acute monocytic leukemia and U937 cells for 24 hours (n=3 technical triplicates). Then, activation of eGFP+ (as a surrogate for CAR+) Jurkat cells was assessed by flow cytometry via staining for the T cell activation marker CD69. Co-culture of CLL-1 CAR expressing Jurkat cells with U937 cells increased CD69$^+$ percentage more compared to THP-1 cells. Statistical analysis was performed by multiple paired t-tests.

(B) Same experiment as in (A): Co-culture of CLL-1 CAR expressing Jurkat cells with both THP-1 and U937 cell lines led to a shift in MFI for CD69.
