# Supplementary material for: Cellular immunotherapy targeting CLL-1 for juvenile myelomonocytic leukemia
Source: Nat Commun. 2025 Apr 23;16:3804. doi: 10.1038/s41467-025-59040-6 (PMC12019388; doi:10.1038/s41467-025-59040-6)
Supplement: Supplementary file 2 — Description of Additional Supplementary Files [file 41467_2025_59040_MOESM2_ESM.pdf]

Description of Additional Supplementary Files

Supplementary Data 1: Sample information for bulk RNAseq.

Sample information on JMML and healthy controls from bulk RNAseq including clinical, laboratory and sequencing data.

Supplementary Data 2: DEG analysis from bulk RNAseq.

DEG analysis for cell surface and non-cell surface proteins from bulk RNAseq and respective expression in GTEx database.

Supplementary Data 3: Sample information for scRNAseq.

Clinical, laboratory and sequencing information of JMML and healthy control samples used in this study for scRNAseq.

Supplementary Data 4: DEG analysis from scRNAseq.

DEG analysis for cell surface and non-cell surface proteins from scRNAseq and respective expression in GTEx database.

Supplementary Data 5: Pathway analysis on HSPCs from scRNAseq.

Pathway analysis for cell surface and non-cell surface proteins on HSPCs from scRNAseq.

Supplementary Data 6: Sample information for mass spectrometry on CD34+ cells.

Sample information on JMML and healthy controls from CD34+ mass spectrometry including clinical, laboratory and sequencing data.

Supplementary Data 7: DEG from mass spectrometry of surface proteins comparing JMML versus healthy control CD34+ cells.

DEG of cell surface annotated proteins comparing JMML and healthy control stem cells and respective expression in GTEx database.

Supplementary Data 8: Sample information for flow cytometry for CLL-1 and in vitro experiments.

Sample information on JMML and healthy controls from flow cytometry including clinical, laboratory and sequencing data.

Supplementary Data 9: Sample information for PDX.

JMML sample information used for PDX including clinical, laboratory and sequencing data.

Supplementary Data 10: Flow cytometry antibodies used to assess primary JMML samples for CLL-1 and CD33 expression.

Information on flow cytometry antibodies including antigen, fluorophore, clone and dilution used.

Supplementary Data 11: Flow cytometry antibodies used for PDX studies.

Information on flow cytometry antibodies including antigen, fluorophore, clone and dilution used.

Supplementary Data 12: Additional flow cytometry antibodies used for other experiments.

Information on flow cytometry antibodies including antigen, fluorophore, clone and dilution used.

Supplementary Data 13: Summarized characteristics of JMML patients and healthy controls for RNAseq, mass spectrometry and flow cytometry.

Clinical, laboratory and sequencing information of JMML and healthy control samples used in this study.

Supplementary Data 14: Specific gene markers used for identification of cell subpopulations by ScType.
