# Supplementary material for: Cellular immunotherapy targeting CLL-1 for juvenile myelomonocytic leukemia
Source: Nat Commun. 2025 Apr 23;16:3804. doi: 10.1038/s41467-025-59040-6 (PMC12019388; doi:10.1038/s41467-025-59040-6)
Supplement: Supplementary file 4 — Reporting Summary [file 41467_2025_59040_MOESM4_ESM.pdf]

nature portfolio

Corresponding author(s): Elliot Stieglitz
Last updated by author(s): 10/15/2024

Reporting Summary

Nature Portfolio wishes to improve the reproducibility of the work that we publish. This form provides structure for consistency and transparency in reporting. For further information on Nature Portfolio policies, see our Editorial Policies and the Editorial Policy Checklist.

Statistics

For all statistical analyses, confirm that the following items are present in the figure legend, table legend, main text, or Methods section.

n/a	Confirmed	
[ ]	[x]	The exact sample size (*n*) for each experimental group/condition, given as a discrete number and unit of measurement
[ ]	[x]	A statement on whether measurements were taken from distinct samples or whether the same sample was measured repeatedly
[ ]	[x]	The statistical test(s) used AND whether they are one- or two-sided<br>*Only common tests should be described solely by name; describe more complex techniques in the Methods section.*
[x]	[ ]	A description of all covariates tested
[ ]	[x]	A description of any assumptions or corrections, such as tests of normality and adjustment for multiple comparisons
[ ]	[x]	A full description of the statistical parameters including central tendency (e.g. means) or other basic estimates (e.g. regression coefficient) AND variation (e.g. standard deviation) or associated estimates of uncertainty (e.g. confidence intervals)
[ ]	[x]	For null hypothesis testing, the test statistic (e.g. *F*, *t*, *r*) with confidence intervals, effect sizes, degrees of freedom and *P* value noted<br>*Give P values as exact values whenever suitable.*
[x]	[ ]	For Bayesian analysis, information on the choice of priors and Markov chain Monte Carlo settings
[x]	[ ]	For hierarchical and complex designs, identification of the appropriate level for tests and full reporting of outcomes
[ ]	[x]	Estimates of effect sizes (e.g. Cohen's *d*, Pearson's *r*), indicating how they were calculated

Our web collection on statistics for biologists contains articles on many of the points above.

Software and code

Policy information about availability of computer code

Data collection	Mass spectrometry: timsTOF pro2 system. Cytokine analysis: Luminex xMAP technology. Live cell analysis: Incucyte. Luciferase assays: Tecan infinite m200 pro. RNAseq: Illumina HiSeq 2000/2500. Flow cytometry: LSRII and Attune (BD FACSDiva, Attune Cytometric Software). In vivo bioluminescence: Living Image (Xenogen).
Data analysis	Flow cytometry: FlowJo v10.10.0. Code for RNAseq: https://github.com/ahdee/StieglitzLab_Werner/. Plots/statistics: also GraphPad Prism V10. SnapGene. In vivo bioluminescence: Living Image (Xenogen). Mass spectrometry: Fragpipe 20.0 (MSFragger v4.0, IonQuant v1.10.12, Philosophor v5.1.0), Metaboanalyst 5.0.

For manuscripts utilizing custom algorithms or software that are central to the research but not yet described in published literature, software must be made available to editors and reviewers. We strongly encourage code deposition in a community repository (e.g. GitHub). See the Nature Portfolio guidelines for submitting code & software for further information.

Data

Policy information about availability of data

All manuscripts must include a data availability statement. This statement should provide the following information, where applicable:

- Accession codes, unique identifiers, or web links for publicly available datasets
- A description of any restrictions on data availability
- For clinical datasets or third party data, please ensure that the statement adheres to our policy

All JMML next-generation sequencing data generated and analyzed during the current study have been deposited in the database of Genotypes and Phenotypes

(dbGaP) under accession phs002504.v3.p3 (UCSF Database for the Advancement of JMML) (https://www.ncbi.nlm.nih.gov/projects/gap/cgi-bin/study.cgi?study_id=phs002504.v3.p3). The RNAseq from healthy controls generated for this study can be obtained by contacting the corresponding author. The mass spectrometry proteomics data have been deposited to the ProteomeXchange Consortium via the PRIDE partner repository with the dataset identifier PXD052910 (https://www.ebi.ac.uk/pride/archive/projects/PXD052910).
The five cord CB HSC bulk RNAseq samples used in this study are available in the GEO database under accession code GSE1118956 (https://www.ncbi.nlm.nih.gov/geo/query/acc.cgi?acc=GSE111895). The three external scRNAseq control samples used in this study are available in dbGaP under accession code phs002371.v6.p1 (https://www.ncbi.nlm.nih.gov/projects/gap/cgi-bin/study.cgi?study_id= phs002371.v6.p1).
The GTEx publicly available data used in this study are available on the GTEx website (file GTEx_Analysis_2017-06-05_v8_RNASeQCv1.1.9_gene_tpm on https://www.gtexportal.org/home/downloads/adult-gtex/bulk_tissue_expression).
The remaining data are available within the Article, Supplementary Information or Source Data that are provided with this paper.

Research involving human participants, their data, or biological material

Policy information about studies with human participants or human data. See also policy information about sex, gender (identity/presentation), and sexual orientation and race, ethnicity and racism.

Reporting on sex and gender	There is a known predisposition for males to develop JMML compared to females with a 2.5:1 male:female ratio although the reason for this imbalance is not known. We report on patients' sex in the main article and in the supplement. JMML is a ultra rare disease and all samples available were included irrespective of (assigned) sex.
Reporting on race, ethnicity, or other socially relevant groupings	The patient samples used in this study were de-identified.
Population characteristics	Patients are all less than 18 years of age and were diagnosed with JMML. Details about genotypic information are meticulously described in the supplemental data.
Recruitment	Patient samples were collected through an IRB approved tissue bank study specifically for JMML. It is possible that patients with more aggressive disease were preferentially referred from outside centers.
Ethics oversight	All the patient samples used in this study were obtained under IRB-approved protocols by the UCSF Committee on Human Research and following the Declaration of Helsinki.

Note that full information on the approval of the study protocol must also be provided in the manuscript.

Field-specific reporting

Please select the one below that is the best fit for your research. If you are not sure, read the appropriate sections before making your selection.

- [x] Life sciences
- [ ] Behavioural & social sciences
- [ ] Ecological, evolutionary & environmental sciences

For a reference copy of the document with all sections, see nature.com/documents/nr-reporting-summary-flat.pdf

Life sciences study design

All studies must disclose on these points even when the disclosure is negative.

Sample size	No statistical methods were used to predetermine sample size. All the quantitative experiments were aimed to be performed with n≥3 and with multiple technical and biological replicates as stated in the legends. Sample sizes were estimated based on previously published results and preliminary experiments. We made the effort to achieve a minimum sample size which proved to be sufficient to reproducibly observe statistically significant differences. Primary patient sample sizes were based on availability of these rare samples.
Data exclusions	Results show all data points collected from the experiments shown in the manuscript.
Replication	Multiple biological and/or technical replicated were performed for experiments unless stated otherwise, and are noted in the figure legends. All experimental findings were reproducible as indicated by the statistical analysis in the figures.
Randomization	For all in vivo experiments, mice were randomized to ensure equally distributed tumor burden in each group before T cells were transferred. For in vitro experiments, randomizing was not required as each experimental condition was controlled within each T cell donor and randomization is not applicable to the reported design.
Blinding	For all in vivo experiments, mouse randomization, injections, monitoring and bioluminescence imaging of the mice were always performed by a blinded member of the Preclinical Therapeutics Core or a blinded member of the Stieglitz laboratory. For tissue processing of euthanized mice and subsequent data collection the investigators were not blinded to the groups but measured with objective methodologies (flow cytometry). For all other in vitro experiments the data collection was not blinded but also measured objectively (e.g. Incucyte, RNAseq). Fully blinded in vitro experiments were not possible due to sample preparation and personnel availability to accomodate such situations.

Reporting for specific materials, systems and methods

We require information from authors about some types of materials, experimental systems and methods used in many studies. Here, indicate whether each material, system or method listed is relevant to your study. If you are not sure if a list item applies to your research, read the appropriate section before selecting a response.

Materials & experimental systems

n/a	Involved in the study	
	x	Antibodies
	x	Eukaryotic cell lines
x		Palaeontology and archaeology
	x	Animals and other organisms
x		Clinical data
x		Dual use research of concern
x		Plants

Methods

n/a	Involved in the study	
x		ChIP-seq
	x	Flow cytometry
x		MRI-based neuroimaging

Antibodies

Antibodies used

This information can be found in Supplementary Data 10-12.

Validation

Information on validation can be found on the following websites of the vendor:
- CD34 AF488 BioLegend (Cat. 343620): https://www.biolegend.com/en-us/products/alexa-fluor-488-anti-human-cd34-antibody-13791
- CD38 BV421 BD Biosciences (Cat. 562444): https://www.bdbiosciences.com/en-us/products/reagents/flow-cytometry-reagents/research-reagents/single-color-antibodies-ruo/bv421-mouse-anti-human-cd38.562444
- CLL-1 APC BioLegend (Cat. 353605): https://www.biolegend.com/de-de/products/apc-anti-human-cd371-clec12a-antibody-9756
- hCD45 APC Fire 750 BioLegend (Cat. 368518): https://www.biolegend.com/de-de/products/apc-fire-750-anti-human-cd45-antibody-13178
- CD33 PE BioLegend (Clone P67.6): https://www.biolegend.com/de-de/products/pe-anti-human-cd33-antibody-12158
- CLL-1 isotype APC BioLegend (Cat. 400219): https://www.biolegend.com/en-us/products/apc-mouse-igg2a-kappa-isotype-ctrl-1397
- CD14 PECy7 BioLegend (Cat. 325618): https://www.biolegend.com/en-us/products/pe-cyanine7-anti-human-cd14-antibody-3958
- CD3 PE BioLegend (Cat. 300308): https://www.biolegend.com/en-us/products/pe-anti-human-cd3-antibody-753
- CD33 BV421 BioLegend (Cat. 303416): https://www.biolegend.com/en-us/products/brilliant-violet-421-anti-human-cd33-antibody-7331
- CD8 PE BioLegend (Cat. 344706): https://www.biolegend.com/en-us/products/pe-anti-human-cd8-antibody-6247
- CD38 BV650 BioLegend (Cat. 356620): https://www.biolegend.com/en-us/products/brilliant-violet-650-anti-human-cd38-antibody-12177
- CD34 BV785 BioLegend (Cat. 343626): https://www.biolegend.com/en-us/products/brilliant-violet-785-anti-human-cd34-antibody-13790
- CD14 BV711 BD Biosciences (Cat. 563373): https://www.bdbiosciences.com/en-us/products/reagents/flow-cytometry-reagents/research-reagents/single-color-antibodies-ruo/bv711-mouse-anti-human-cd14.563373
- CD11b PECy7 BioLegend (Cat. 301322): https://www.biolegend.com/en-us/products/pe-cyanine7-anti-human-cd11b-antibody-3940
- anti-human lineage cocktail APC BioLegend (Cat. 348803): https://www.biolegend.com/en-us/products/apc-anti-human-lineage-cocktail-cd3-cd14-cd16-cd19-cd20-cd56-8073
- mCD45.1 BV421 BioLegend (Cat. 103134): https://www.biolegend.com/en-us/products/brilliant-violet-421-anti-mouse-cd45-antibody-7253
- CD107a APC BD Biosciences (Cat. 560664): https://www.bdbiosciences.com/en-us/products/reagents/flow-cytometry-reagents/research-reagents/single-color-antibodies-ruo/apc-mouse-anti-human-cd107a.560664
- CD69 APC BD Biosciences (Cat. 560711): https://www.bdbiosciences.com/en-us/products/reagents/flow-cytometry-reagents/research-reagents/single-color-antibodies-ruo/apc-mouse-anti-human-cd69.560711
- CD25 BV510 BD Biosciences (Cat. 563351): https://www.bdbiosciences.com/en-us/products/reagents/flow-cytometry-reagents/research-reagents/single-color-antibodies-ruo/bv510-mouse-anti-human-cd25.563351
- CD45RA BV605 BioLegend (Cat. 304134): https://www.biolegend.com/en-us/products/brilliant-violet-605-anti-human-cd45ra-antibody-7661
- CD62L PE BioLegend (Cat. 304840): https://www.biolegend.com/en-us/products/pe-anti-human-cd62l-antibody-653
- CD3 PerCP BioLegend (Cat. 300326): https://www.biolegend.com/en-us/products/percp-anti-human-cd3-antibody-5612
- LAG3 APC BioLegend (Cat. 369212): https://www.biolegend.com/en-us/products/apc-anti-human-cd223-antibody-15464
- TIM3 PE BioLegend (Cat. 345006): https://www.biolegend.com/en-us/products/pe-anti-human-cd366-tim-3-antibody-6121
- PD1 BV605 BioLegend (Cat. 367426): https://www.biolegend.com/en-us/products/pe-anti-human-cd366-tim-3-antibody-6121

Eukaryotic cell lines

Policy information about cell lines and Sex and Gender in Research

Cell line source(s)	THP-1 (TIB-202), HL-60 (CCL-240), U937 (CRL-1593.2), K562 (CCL-243), Jurkat (TIB-152), 293T (CRL-3216) cells: ATCC Luciferase mCherry expressing cell lines: generated in this study
Authentication	COA were provided with cell lines from ATCC. Relevant antigen expression was routinely confirmed by flow cytometry. THP-1, U937 and Jurkat cells were authenticated by STR analysis.
Mycoplasma contamination	Jurkat cells were tested negative for mycoplasma contamination using Lookout Mycoplasma PCR Detection kit (Sigma-Aldrich, MP0035-1KT). The remaining cell lines were not tested.
Commonly misidentified lines (See ICLAC register)	ICLAR register was assessed and no commonly misidentified lines were used.

Animals and other research organisms

Policy information about studies involving animals; ARRIVE guidelines recommended for reporting animal research, and Sex and Gender in Research

Laboratory animals	Male or female NSG, NSG-SGM3 and NOG-EXL mice were used (6-12 weeks old) and either obtained from breeding colonies maintained by UCSF or bought from JAX lab or Taconic. Mice allocated to different experimental groups were sex-matched unless otherwise indicated.
Wild animals	This study did not involve wild animals.
Reporting on sex	Sex considerations were beyond the scope of the study design.
Field-collected samples	No field collected samples were used.
Ethics oversight	Mice were used in accordance with guidelines established by the Institutional Animal Care and Use Committee (IACUC) and Laboratory Animal Resource Center (LARC) at UCSF (application AN201693). Humane endpoint was not exceeded.

Note that full information on the approval of the study protocol must also be provided in the manuscript.

Plants

Seed stocks	Not applicable
Novel plant genotypes	Not applicable
Authentication	Not applicable

Flow Cytometry

Plots

Confirm that:

- [x] The axis labels state the marker and fluorochrome used (e.g. CD4-FITC).
- [x] The axis scales are clearly visible. Include numbers along axes only for bottom left plot of group (a 'group' is an analysis of identical markers).
- [x] All plots are contour plots with outliers or pseudocolor plots.
- [x] A numerical value for number of cells or percentage (with statistics) is provided.

Methodology

Sample preparation	Cells were cell lines, (CAR) T cells, primary leukemia samples or mouse tissue. Murine spleens were disaggregated with the back of a syringe plunger and passed through a 70 um cell strainer. Murine leg bones were spun 5 min at 11,000 g to collect cells. Murine blood and bone marrow were treated with ACK buffer or BD Lysing solution for red blood cell lysis. They were labeled at 4°C after blockade of Fc receptors. Antibodies are listed in Supplementary Data Table 10-12. Brilliant Stain Buffer Plus (BD Biosciences, 566385) was added when using multiple Brilliant Violet antibodies. Antibodies were titrated using the staining index. Compensation was performed using BD CompBeads (BD Biosciences, 51-90-9001229), ArC reactive beads (Life Technologies, 2480653) and GFP BrightComp Beads (Life Technologies, A10514). The buffer used contained D-PBS, 2% FBS and ethylenediaminetetraacetic acid (EDTA).
Instrument	LSRII (BD Biosciences) or Attune Nxt (Thermo Fisher Scientific)
Software	BD FACSDiva, Attune Cytometric Software
Cell population abundance	For the injection of CD34+CD38- CLL-1+ vs. CLL-1- cells, cell population abundance ranged between 0-0.9%. The purity of post-sort fractions was not determined due to the rarity of the sorted cells and needed cell numbers for experiments.
Gating strategy	Cells of interest (debris exclusion) in FSC-A/SSC-A gate -> doublet exclusion in FSC-A/FSC-H except debris -> slive dead gate if applicable -> sequential surface marker gates. Boundaries drawn based on unstained, positive and FMO controls. Further information on gating strategies for specific experiments can be found in the figures in the Supplementary Information.

- [x] Tick this box to confirm that a figure exemplifying the gating strategy is provided in the Supplementary Information.
